# Bronchial thermoplasty in asthma: an exploratory histopathological evaluation in distinct asthma endotypes/phenotypes

**DOI:** 10.1186/s12931-021-01774-0

**Published:** 2021-06-28

**Authors:** Eleni Papakonstantinou, Triantafyllia Koletsa, Liang Zhou, Lei Fang, Michael Roth, Meropi Karakioulaki, Spasenija Savic, Leticia Grize, Michael Tamm, Daiana Stolz

**Affiliations:** 1 Clinic of Respiratory Medicine and Pulmonary Cell Research, University Hospital of Basel and Department of Biomedicine, University of Basel, Petersgraben 4, 4031 Basel, Switzerland; 2 Laboratory of Pharmacology, Faculty of Medicine, Aristotle University of Thessaloniki, Thessaloniki, Greece; 3 Department of Pathology, Faculty of Medicine, Aristotle University of Thessaloniki, Thessaloniki, Greece; 4 Department of Pathology, University Hospital of Basel, Basel, Switzerland

**Keywords:** Bronchial thermoplasty, Severe asthma, Asthma endotypes, Asthma phenotypes, Heat shock proteins, Glucocorticoid receptor, Epithelial cell regeneration, Airway smooth muscle

## Abstract

**Background:**

Bronchial thermoplasty regulates structural abnormalities involved in airway narrowing in asthma. In the present study we aimed to investigate the effect of bronchial thermoplasty on histopathological bronchial structures in distinct asthma endotypes/phenotypes.

**Methods:**

Endobronchial biopsies (n = 450) were collected from 30 patients with severe uncontrolled asthma before bronchial thermoplasty and after 3 sequential bronchial thermoplasties. Patients were classified based on blood eosinophils, atopy, allergy and smoke exposure. Tissue sections were assessed for histopathological parameters and expression of heat-shock proteins and glucocorticoid receptor. Proliferating cells were determined by Ki67-staining.

**Results:**

In all patients, bronchial thermoplasty improved asthma control (p < 0.001), reduced airway smooth muscle (p = 0.014) and increased proliferative (Ki67 +) epithelial cells (p = 0.014). After bronchial thermoplasty, airway smooth muscle decreased predominantly in patients with T2 high asthma endotype. Epithelial cell proliferation was increased after bronchial thermoplasty in patients with low blood eosinophils (p = 0.016), patients with no allergy (p = 0.028) and patients without smoke exposure (p = 0.034).

In all patients, bronchial thermoplasty increased the expression of glucocorticoid receptor in epithelial cells (p = 0.018) and subepithelial mesenchymal cells (p = 0.033) and the translocation of glucocorticoid receptor in the nucleus (p = 0.036). Furthermore, bronchial thermoplasty increased the expression of heat shock protein-70 (p = 0.002) and heat shock protein-90 (p = 0.001) in epithelial cells and decreased the expression of heat shock protein-70 (p = 0.009) and heat shock protein-90 (p = 0.002) in subepithelial mesenchymal cells. The effect of bronchial thermoplasty on the expression of heat shock proteins -70 and -90 was distinctive across different asthma endotypes/phenotypes.

**Conclusions:**

Bronchial thermoplasty leads to a diminishment of airway smooth muscle, to epithelial cell regeneration, increased expression and activation of glucocorticoid receptor in the airways and increased expression of heat shock proteins in the epithelium. Histopathological effects appear to be distinct in different endotypes/phenotypes indicating that the beneficial effects of bronchial thermoplasty are achieved by diverse molecular targets associated with asthma endotypes/phenotypes.

**Supplementary Information:**

The online version contains supplementary material available at 10.1186/s12931-021-01774-0.

## Background

Asthma is now considered an umbrella diagnosis for pathologies with distinct mechanistic pathways (endotypes) and clinical presentations (phenotypes) [1]. These endotypes and phenotypes are key elements for precision medicine in the heterogeneous asthma profiles.

Bronchial thermoplasty is a non-pharmacological treatment for severe asthma. It is based on selective heating of the airways using a bronchoscope-inserted catheter that ends in 4 electrodes and generates a temperature of 65 °C for 10 s [2]. This procedure aims to improve asthma symptoms by targeting structural components of the airways.

In previous clinical trials, it was demonstrated that bronchial thermoplasty was associated with a reduction in the number of exacerbations in severe asthmatics, a step-down in treatment and an improved quality of life without improvement of lung function [3, 4]. Integrated in vitro and in silico modelling suggested that the reduction in airway smooth muscle cells (ASMC) after bronchial thermoplasty cannot be fully explained by acute heating and furthermore, it could not confer the great improvement in asthma control [5]. Tο this end, it was suggested that the heat energy that is produced during bronchial thermoplasty can alter airway structural components other than ASMC that are involved in airway narrowing and bronchial reactivity such as neuroendocrine epithelial cells and nerve endings [6]. Histological analysis of endobronchial biopsies is an important tool to ascertain the constitution of the epithelial and mesenchymal bronchial compartments that may reflect underlying inflammatory processes (endotypes) which translate in diverse clinical presentations (phenotypes) [7, 8].

Response to inhaled glucocorticoids depends on the presence of the glucocorticoid receptor (GR) in the correct conformation, that is only achieved when GR forms a complex with heat shock proteins (HSP) 70 and HSP90 [9]. HSPs comprise a family of stress-response proteins that are induced under threatening alterations of the cellular environment [10]. An important role of HSPs is that they bind to GR intracellularly and change its structure into a conformation that exhibits a high-affinity for steroid binding. We have previously shown that the expression of HSP60 is altered in bronchoalveolar lavage of patients with severe asthma after bronchial thermoplasty and that specific HSPs are involved in intracellular pathways associated with airway remodeling [11]. In addition, exposure to heat modified the expression of HSP70 and HSP90 in a cell-type specific manner, indicating that the regenerative potential of the epithelium is increased by bronchial thermoplasty, while that of ASMC is reduced [12].

The current exploratory, hypothesis-generating study aimed to evaluate the effect of bronchial thermoplasty on histopathological findings in asthma patients with different endotypes such as eosinophilia and phenotypes such as atopy, allergy and relevant smoke exposure. We hypothesized that histopathological alterations after bronchial thermoplasty differ between asthma patients with distinct endotypes/phenotypes.

## Methods

### Patients, bronchial thermoplasty

This is a prospective, monocentric, observational study including 30 patients diagnosed with severe asthma based on ERS/ATS guidelines and GINA 2017 criteria, who fulfilled the indication for bronchial thermoplasty as they all had a symptomatic disease with severe, persistent, poorly-controlled symptoms, recurrent exacerbations, emergency department visits and hospitalizations despite maximal medical treatment. Exclusion criteria were: (a) pulmonary condition other than asthma as the main respiratory disease, e.g., bronchiectasis; (b) decrease in diffusion capacity (as defined by a cDLCO < 50%); (c) rapid lethal disease, e.g., bronchial carcinoma, advance heart failure, end-stage renal failure; (d) severe immunosuppression including manifested AIDS, organ transplantation or neutropenia (< 500 × 10^9^/L).

All patients were prospectively classified in the following endotypes/phenotypes: (1) patients with high (≥ 300/μl) and low (< 300/μl) eosinophils; (2) patients with atopy (IgE ≥ 100 U/ml) and without atopy (IgE < 100 U/ml); (3) patients with allergy (positive prick test) and without allergy (negative prick test); (4) patients with relevant smoke exposure (≥ 15 pack-years) and without relevant smoke exposure (< 15 pack-years).

The study was performed according to the GCP guidelines and was approved by the local Institutional Review Board (EKNZ 2016-01057). All patients provided written informed consent to participate in the study.

All patients underwent three sessions of bronchial thermoplasty separated by at least 1-month intervals [13–16]. During the procedure, EBB specimens were obtained from first- and second- generation bronchi using 2.2 mm wide single use biopsy forceps with Endo-Glide Sheath (Radial Jaw, Boston Scientific). All EBB specimens were washed in PBS, fixed in formalin and transferred to pathology (Additional file 1: Figure S1).

### Histological evaluation, immunohistochemistry

To evade dissimilarities in the histology between different lobes, [17] we performed analysis of EBB (n = 3–5) of individual patients obtained from the right lower lobe before and after each bronchial thermoplasty. For each specimen, 5 sequential sections were stained with Hematoxylin/Eosin and Elastica van Gieson and were evaluated blindly by 2 senior pathologists. Only specimens with tangential sections were evaluated, so that the orientation of the sections would not affect the measurements (Additional File 1: Figure S1).

Inflammation in the stroma, tissue lymphocyte/ eosinophil/ granulocyte infiltration and thickening of reticular basement membrane (BM), were appraised using a 0–3 scale: 0 = absence/normal, 1 = mild-moderate, 2–3 = severe. The median value of all assessments for these categorical measurements was assigned to each patient as follows: 0 < 0.5 = (absence/normal), 0.5 < 1.50 = mild-moderate, 1.5–3 = severe.

Airway smooth muscle (ASM) mass was evaluated as the total percentage of the submucosal area occupied by ASMC. The distance between BM and ASM in μm, was measured from the parenchymal site of the BM towards the ASM, without including the thickness of the BM. The mean values of all assessments for these numerical measurements were assigned to each patient.

EBB tissue sections obtained before and after bronchial thermoplasty were stained using antigen-specific antibodies for Ki67, GR, HSP70 and HSP90 (Additional file 1: Figure S1).

### Statistical analysis

Patient’s characteristics were summarized as means and standard errors if continuous and as counts and percentages if categorical. Comparisons between endotypes/phenotypes were carried out using the Fisher’s exact test or Mann–Whitney U-test.

Comparison of outcomes obtained before and after bronchial thermoplasty were performed using rank mixed linear regression models, if continuous, and mixed multinomial (ordinal) logistic regression models if categorical. In both cases, the factor patient was included as a random effect.

Statistics were calculated using the softwares SAS (v. 9.4, 2012, SAS Institute Inc. Cary, NC, USA) and SPSS (Windows version 23.0).

## Results

### Patients, asthma control after thermoplasty

**Fig. 1:**
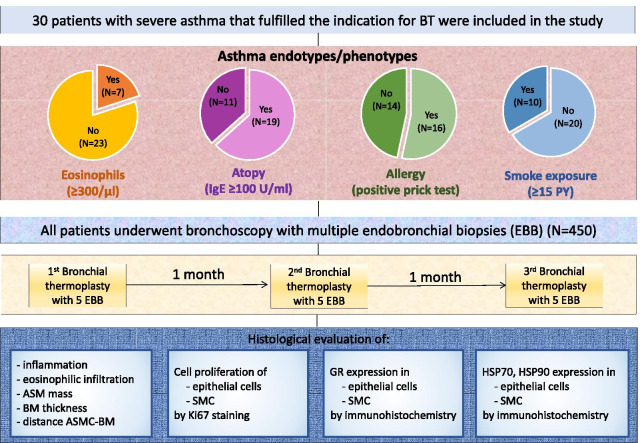
The CONSORT flow diagram of the study. All 30 asthma patients fulfilled the criteria for bronchial thermoplasty as they all had persistent, poorly-controlled symptoms, recurrent exacerbations, emergency department visits and hospitalizations despite maximal medical treatment. *PY* pack years, *BM* basement membrane, *ASM* airway smooth muscle, *ASMC* airway smooth muscle cells, *SMC* subepithelial mesenchymal cells, *EBB* endobronchial biopsies

**Fig. 2:**
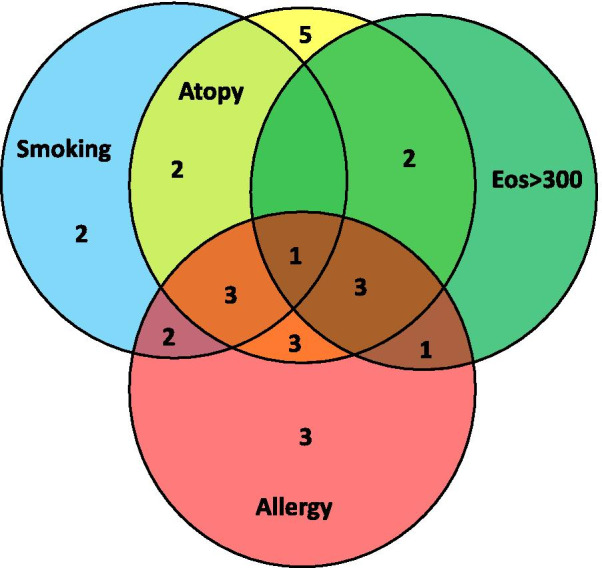
Venn diagram that depicts overlaps between different endotypes/phenotypes. Patients with relevant smoke exposure: N = 20; patients with allergy: N = 16; patients with atopy: N = 19; patients with eosinophils ≥ 300 μl: N = 7

The mean age of the 30 asthma patients was 57.8 years. The majority (70%) of the patients were classified as GINA 5 and were under treatment with oral corticosteroids (Table 1). Among the 30 asthma patients, seven patients had blood eosinophils ≥ 300/μl and classified to eosinophilic asthma endotype; 10 patients had relevant smoke exposure (≥ 15 PY) and classified to smoking phenotype; 16 patients had positive prick test and classified to allergic phenotype (Fig. 1). However, there were overlaps between the different groups as shown in Fig. 2. Patients of distinct endotypes and phenotypes had similar baseline characteristics (Table 1). Yet, patients with eosinophils < 300/μl had significantly lower FEV1/FVC as compared with patients with eosinophils ≥ 300/μl (p = 0.042). Furthermore, as compared to patients without smoke exposure, patients with relevant smoke exposure had lower, FEV1/FVC (p = 0.022), and higher RV% predicted (p = 0.028) and TLC% predicted (p = 0.007).

**Table 1:** Characteristics of the asthma patients included in the study

Parameters	All patients (N = 30)	Asthma endotypes/phenotypes											
		Eos ≥ 300/μl (N = 7)	Eos < 300/μl (N = 23)	P value*	Atopy (N = 19)	No Atopy (N = 11)	P value*	Allergy (N = 16)	No allergy (N = 14)	P value*	Smoke exposure (N = 10)	No smoke exposure (N = 20)	P value*
Age, years, mean ± SEM	57.8 ± 2.9	50.1 ± 6.1	60.1 ± 3.1	0.128^b^	57.4 ± 3.9	58.4 ± 4.0	0.832^b^	53.5 ± 4.3	62.6 ± 3.3	0.176^b^	63.0 ± 1.9	55.2 ± 4.1	0.108^b^
Gender, Female/Male	19/11	5/2	14/9	1.000^a^	11/8	8/3	0.466^a^	12/4	7/7	0.257^a^	5/5	14/6	0.425^a^
BMI, mean ± SEM	27.8 ± 0.9	29.7 ± 2.7	27.2 ± 0.9	0.202^b^	27.4 ± 1.2	28.5 ± 1.4	0.714^b^	28.3 ± 1.4	27.2 ± 1.1	0.533^b^	28.4 ± 1.1	27.5 ± 1.3	0.644^b^
Pack Years, mean ± SEM	14.0 ± 4.2	8.3 ± 4.4	15.6 ± 5.1	0.653^b^	12.5 ± 5.5	16.8 ± 5.9	0.342^b^	16.3 ± 6.5	11.4 ± 4.7	0.622^b^	31.5 ± 8.5	4.3 ± 2.6	< 0.001^b^
Oral corticosteroids, n (%)	21 (70.0)	4 (57.1)	17 (73.9)	0.640^a^	13 (68.4)	8 (72.7)	1.000^a^	11 (68.8)	10 (71.4)	1.000^a^	7 (70.0)	14 (70.0)	1.000^a^
Oral corticosteroids, mg, mean ± SEM	13.8 ± 1.5	16.3 ± 5.5	13.2 ± 1.5	0.171^b^	14.4 ± 1.8	12.8 ± 2.9	0.453^b^	12.3 ± 2.4	15.5 ± 1.9	0.201^b^	12.5 ± 2.7	14.4 ± 1.9	0.511^b^
FEV1% of predicted value, mean ± SEM	66.9 ± 3.6	74.5 ± 6.1	64.6 ± 4.3	0.111^b^	68.0 ± 5.0	65.1 ± 4.9	0.863^b^	64.3 ± 4.6	69.9 ± 5.6	0.662^b^	62.8 ± 8.0	69.0 ± 3.7	0.281^b^
FVC % of predicted value, mean ± SEM	94.7 ± 3.5	93.3 ± 8.1	95.1 ± 3.9	0.713^b^	95.5 ± 4.5	93.3 ± 5.8	0.983^b^	92.4 ± 5.3	97.4 ± 4.4	0.506^b^	101.5 ± 4.9	91.3 ± 4.5	0.248^b^
FEV1/FVC, mean ± SEM	73.0 ± 3.2	84.2 ± 5.7	69.6 ± 3.6	0.042^b^	73.7 ± 4.5	71.8 ± 4.5	0.880^b^	72.5 ± 4.3	73.6 ± 5.0	0.835^b^	62.8 ± 6.0	78.1 ± 3.4	0.022^b^
RV % of predicted value, mean ± SEM	118.5 ± 6.8	103.8 ± 16.2	123.0 ± 7.4	0.249^b^	118.7 ± 9.8	118.2 ± 8.4	0.813^b^	123.7 ± 10.5	112.5 ± 8.5	0.480^b^	139.7 ± 11.2	107.9 ± 7.7	0.028^b^
TLC % of predicted value, mean ± SEM	104.3 ± 3.1	98.3 ± 8.5	106.1 ± 3.2	0.364^b^	105.0 ± 4.5	103.1 ± 3.8	0.651^b^	105.4 ± 4.6	103.0 ± 4.2	0.835^b^	116.3 ± 3.5	98.3 ± 3.7	0.007^b^
RV/TLC, mean ± SEM	108.6 ± 4.2	99.5 ± 8.9	111.4 ± 4.7	0.315^b^	106.1 ± 5.0	113.1 ± 7.5	0.621^b^	112.1 ± 6.5	104.7 ± 5.1	0.383^b^	112.6 ± 6.5	106.6 ± 5.4	0.481^b^
DLCO % of predicted value, mean ± SEM	89.1 ± 3.8	89.1 ± 7.6	89.2 ± 4.5	0.787^b^	88.8 ± 4.5	89.7 ± 7.2	0.813^b^	88.1 ± 5.5	90.4 ± 5.4	0.835^b^	87.8 ± 6.6	89.8 ± 4.8	0.538^b^
Reversibility FEV1%, mean ± SEM	12.0 ± 1.9	6.8 ± 2.0	13.6 ± 2.3	0.126^b^	11.5 ± 2.1	12.6 ± 3.5	0.859^b^	13.6 ± 2.5	10.4 ± 2.8	0.275^b^	10.2 ± 2.0	12.8 ± 2.6	0.601^b^
FeNO ppm, mean ± SEM	37.9 ± 5.9	53.1 ± 15.6	33.3 ± 5.9	0.169^b^	38.8 ± 7.2	36.4 ± 10.4	0.366^b^	36.4 ± 7.5	39.6 ± 9.5	0.803^b^	34.4 ± 8.2	39.7 ± 7.9	0.775^b^
IgE U/ml, mean ± SEM	349.5 ± 96.9	492.1 ± 129.5	306.1 ± 120.1	0.053^b^	524.4 ± 138.6	47.4 ± 7.5	< 0.001^b^	508.9 ± 170.1	167.4 ± 43.8	0.244^b^	388.7 ± 249.6	329.9 ± 81.8	0.692^b^

*Comparisons were made by Fisher’s exact test (^a^) or by the Mann–Whitney test (^b^)

*SEM* standard error of the mean, *BMI* body mass index, *FEV*_*1*_ forced expiratory volume in 1 s, *FVC* forced vital capacity, *TLC* total lung capacity, *RV* residual volume, *DLCO* diffusing capacity for carbon monoxide, *FeNO* fractional exhaled NO, *IgE* immunoglobulin E, *Eos* eosinophils

Lung function parameters were assessed before the 1st bronchial thermoplasty, 1 month after each sequential bronchial thermoplasty and at a follow-up visit, 49.65 ± 35.25 days after the 3rd bronchial thermoplasty. There were no significant changes in lung function parameters after serial bronchial thermoplasties (Additional file 2: Table S1). However, Asthma Control Test (ACT) revealed a significant improvement of disease control after bronchial thermoplasty in all patients (p < 0.001) [mean change (SEM) in ACT total score between before bronchial thermoplasty and 3 months after last bronchial thermoplasty of 3.8 (3.9)]. Improvement of ACT total score was similar across distinct asthma endotypes/phenotypes.

### Histological evaluation of endobronchial biopsies

Comparisons using mixed multinomial logistic regression models revealed that inflammation in the stroma, tissue infiltration with lymphocytes, eosinophils and granulocytes and BM thickening were not altered significantly after bronchial thermoplasty in EBB of asthma patients (Table 2A). However, after bronchial thermoplasty, ASM mass was significantly decreased (p = 0.014), while there was no significant difference in the distance between BM and ASMC (Table 2A). Representative microphotographs depicting the decrease of ASM mass in EBB of asthma patients after bronchial thermoplasty are shown in Additional File 1: Figure S1.

**Table 2:** Histopathological evaluation of endobronchial biopsies before and after bronchial thermoplasty

[A]	All asthma patients (N = 30)			
	Before 1st BT	At 2nd BT 1 month after 1st BT	At 3rd BT 1 month after 2nd BT	P Value*
Inflammation in the stroma** Absence, n (%) Mild-moderate, n (%) High, n (%)	Ν = 30 5 (16.7) 15 (50.0) 10 (33.3)	Ν = 27 5 (18.5) 13 (48.2) 9 (33.3)	Ν = 23 4 (17.4) 12 (52.2) 7 (30.4)	**0.948**
Tissue lymphocyte infiltration** Absence, n (%) Mild-moderate, n (%) High, n (%)	Ν = 30 7 (23.3) 12 (40.0) 11 (36.7)	Ν = 27 5 (18.5) 15 (55.5) 7 (25.9)	Ν = 23 4 (17.4) 14 (60.9) 5 (21.7)	**0.814**
Tissue eosinophil infiltration** Absence, n (%) Mild-moderate, n (%) High, n (%)	Ν = 30 14 (46.7) 7 (23.4) 9 (30.0)	Ν = 27 19 (70.3) 3 (11.1) 5 (18.5)	Ν = 23 14 (60.9) 7 (30.4) 2 (8.7)	**0.149**
Granulocytes in the stroma** Absence, n (%) Mild-moderate, n (%) High, n (%)	Ν = 30 19 (63.3) 11 (36.4) 0	Ν = 27 21 (77.8) 6 (22.2) 0	Ν = 23 18 (78.3) 4 (17.4) 1 (4.3)	**0.408**
BM thickening ** Normal, n (%) Mild-moderate, n (%) High, n (%)	Ν = 30 1 (3.3) 19 (63.3) 10 (33.3)	Ν = 25 5 (20.0) 15 (60.0) 5 (20.0)	Ν = 23 2 (8.7) 14 (60.9) 7 (30.4)	**0.148**
Average ASM mass (%) Median [IQR] Mean (SD)	Ν = 30 13.3 [4.8–31.2] 19.0 (17.5)	Ν = 26 4.2 [0.0–15.0] 10.8 (14.8)	N = 23 2.5 [0.0–18.0] 9.6 (16.4)	**0.014**
Distance BM-ASM μm, Median [IQR] Mean (SD)	Ν = 24 73.2 [36.7–117.3] 83.9 (56.1)	N = 11 56.5 [24.9–161.8] 95.0 (86.0)	N = 8 81.6 [35.5–126.4] 95.4 (77.5)	**0.987**

[B]	Asthma patients with blood eosinophils ≥ 300/μL (N = 7)				Asthma patients with blood eosinophils < 300/μL (N = 23)			
	Before 1^st^ BT	At 2nd BT 1 month after 1^st^ BT	At 3rd BT 1 month after 2^nd^ BT	P value*	Before 1st BT	At 2nd BT 1 month after 1st BT	At 3rd BT 1 month after 2nd BT	P value*
Inflammation in the stroma** Absence, n (%) Mild-moderate, n (%) High, n (%)	N = 7 2 (28.6) 2 (28.6) 3 (42.8)	N = 5 1 (20.0) 2 (40.0) 2 (40.0)	N = 5 0 2 (40.0) 3 (60.0)	0.589	N = 23 3 (13.0) 13 (56.5) 7 (30.3)	N = 22 4 (18.2) 11 (50.0) 7 (31.7)	N = 18 4 (22.2) 9 (50.0) 5 (27.9)	0.665
Tissue lymphocyte infiltration** Absence, n (%) Mild-moderate, n (%) High, n (%)	N = 7 1 (14.3) 2 (28.6) 4 (57.1)	N = 5 1 (20.0) 3 (60.0) 1 (20.0)	N = 5 0 4 (80.0) 1 (20.0)	0.279	N = 23 6 (26.1) 10 (43.5) 7 (30.4)	N = 22 4 (18.2) 12 (54.5) 6 (27.1)	N = 18 4 (22.2) 10 (55.5) 4 (22.3)	0.836
Tissue eosinophil infiltration** Absence, n (%) Mild-moderate, n (%) High, n (%)	N = 7 4 (57.1) 1 (14.3) 2 (28.6)	N = 5 3 (60.0) 0 2 (40.0)	N = 5 2 (40.0) 3 (60.0) 0	0.980	N = 23 10 (43.5) 6 (26.0) 7 (30.4)	N = 22 16 (72.7) 3 (13.6) 3 (13.6)	N = 18 12 (66.7) 4 (22.2) 2 (11.2)	0.109
Granulocytes in the stroma** Absence, n (%) Mild-moderate n (%) High, n (%)	N = 7 6 (85.7) 1 (14.3) 0	N = 5 4 (80.0) 1 (20.0) 0	N = 5 4 (80.0) 1 (20.0) 0	0.949	N = 23 13 (56.5) 10 (43.5) 0	N = 22 17 (77.3) 5 (22.7) 0	N = 18 14 (77.8) 3 (16.7) 1 (5.6)	0.280
BM thickening ** Normal, n (%) Mild-moderate, n (%) High, n (%)	N = 7 1 (14.3) 3 (42.8) 3 (42.8)	N = 5 1 (20.0) 2 (40.0) 2 (40.0)	N = 5 1 (20.0) 2 (40.0) 2 (40.0)	0.821	N = 23 1 (4.3) 15 (65.2) 7 (30.4)	N = 20 4 (20.0) 13 (65.0) 3 (15.0)	N = 18 1 (5.6) 12 (66.7) 5 (27.8)	0.126
Average ASM mass (%) Median [IQR] Mean (SD)	N = 7 43.3 [10.5–50.0] 33.3 (20.1)	N = 5 10.0 [5.0–12.5] 9.0 (5.5)	N = 5 1.0 [0.0–10.3] 4.3 (7.7)	0.009	N = 23 12.5 [1.3–20.0] 14.7 (14.4)	N = 21 2.5 [0.0–21.7] 11.2 (16.3)	N = 18 2.5 [0.0–20.0] 10.3 (17.2)	0.248
Distance BM-ASM μm Median [IQR] Mean (SD)	N = 7 67.7 [45.0–79.0] 63.7 (20.1)	N = 1 296.0 296.0	N = 1 114.3 114.3	ǂ	N = 17 76.0 [33.7–136.0] 92.2 (64.3)	N = 10 55.5 [23.7–123.9] 74.9 (57.3)	N = 7 75.8 [32.6–130.4] 92.7 (83.4)	0.770

[C]	Asthma patients with Atopy (N = 19)				Asthma patients without atopy (N = 11)			
	Before 1st BT	At 2nd BT 1 month after 1st BT	At 3rd BT 1 month after 2nd BT	P Value*	Before 1st BT	At 2nd BT 1 month after 1st BT	At 3rd BT 1 month after 2nd BT	P Value*
Inflammation in the stroma** Absence, n (%) Mild-moderate, n (%) Severe, n (%)	N = 19 5 (26.3) 6 (31.6) 8 (42.2)	N = 17 4 (23.5) 7 (41.2) 6 (35.3)	N = 13 2 (15.4) 7 (53.9) 4 (30.8)	0.972	N = 11 0 9 (81.8) 2 (18.2)	N = 10 1 (10.0) 6 (60.0) 3 (30.0)	N = 10 2 (20.0) 5 (50.0) 3 (30.0)	0.942
Tissue lymphocyte infiltration** Absence, n (%) Mild-moderate, n (%) Severe, n (%)	N = 19 4 (21.1) 6 (31.6) 9 (47.4)	N = 17 4 (23.5) 8 (47.1) 5 (29.5)	N = 13 2 (15.4) 8 (61.6) 3 (23.1)	0.516	N = 11 3 (27.3) 6 (54.5) 2 (18.2)	N = 10 1 (10.0) 7 (70.0) 2 (20.0)	N = 10 2 (20.0) 6 (60.0) 2 (20.0)	0.800
Tissue eosinophil infiltration** Absence, n (%) Mild-moderate, n (%) Severe, n (%)	N = 19 10 (52.6) 3 (15.8) 6 (31.6)	N = 17 12 (70.6) 2 (11.8) 3 (17.7)	N = 13 6 (46.2) 6 (46.2) 1 (7.7)	0.420	N = 11 4 (36.4) 4 (36.4) 3 (27.3)	N = 10 7 (70.0) 1 (10.0) 2 (20.0)	N = 10 8 (80.0) 1 (10.0) 1 (10.0)	0.198
Granulocytes in the stroma** Absence, n (%) A few, n (%) Many, n (%)	N = 19 13 (68.4) 6 (31.6) 0	N = 17 13 (76.5) 4 (23.5) 0	N = 13 10 (76.9) 3 (23.1) 0	0.783	N = 11 6 (54.5) 5 (45.5) 0	N = 10 8 (80.0) 2 (20.0) 0	N = 10 8 (80.0) 1 (10.0) 1 (10.0)	0.444
BM thickening ** Normal, n (%) Mild-moderate, n (%) Severe, n (%)	N = 19 1 (5.3) 12 (63.2) 6 (31.6)	N = 15 2 (13.3) 9 (60.0) 4 (26.7)	N = 13 8 (61.5) 0 5 (38.5)	0.522	N = 11 0 7 (63.6) 4 (36.4)	N = 10 3 (30.0) 6 (60.0) 1 (10.0)	N = 10 2 (20.0) 7 (70.0) 1 (10.0)	0.146
Average ASMC mass (%) Median [IQR] Mean (SD)	N = 19 13.3 [1.3–30.0] 18.5 (17.9)	N = 16 4.2 [0.0–11.9] 7.8 (9.8)	N = 13 0.0 [0.0–9.4] 4.8 (7.7)	0.012	N = 11 13.3 [5.0–35.0] 19.9 (17.4)	N = 10 6.3 [0.0–31.3] 15.5 (20.2)	N = 10 4.2 [0.9–25.6] 15.9 (22.4)	0.424
Distance BM-ASMC μm Median [IQR] Mean (SD)	N = 13 79.0 [54.4–119.8] 94.3 (64.7)	N = 5 111.3 [35.3–232.5] 129.4 (109.4)	N = 2 100.8 100.8 (18.9)	0.547	N = 11 71.5 [33.5–124.5] 71.6 (43.8)	N = 6 55.5 [20.5–110.4] 66.4 (55.3)	N = 6 59.9 [28.8–163.0] 93.5 (91.3)	0.896

[D]	Asthma patients with allergy (N = 16)				Asthma patients without allergy (N = 14)			
	Before 1st BT	At 2^nd^ BT 1 month after 1st BT	At 3^rd^ BT 1 month after 2nd BT	P value*	Before 1st BT	At 2^nd^ BT 1 month after 1st BT	At 3^rd^ BT 1 month after 2nd BT	P value*
Inflammation in the stroma** Absence, n (%) Mild-moderate, n (%) High, n (%)	N = 16 3 (18.8) 8 (50.0) 5 (31.2)	N = 14 2 (14.3) 5 (35.7) 7 (50.0)	N = 13 2 (15.4) 7 (53.8) 4 (30.8)	0.588	N = 14 2 (14.3) 7 (50.0) 5 (35.7)	N = 13 3 (23.1) 8 (61.5) 2 (15.4)	N = 10 2 (20.0) 5 (50.0) 3 (30.0)	0.410
Tissue lymphocyte infiltration** Absence, n (%) Mild-moderate, n (%) High, n (%)	N = 16 4 (25.0) 7 (43.7) 5 (31.3)	N = 14 2 (14.3) 7 (50.0) 5 (35.7)	N = 13 2 (15.4) 9 (69.2) 2 (15.4)	0.698	N = 14 3 (21.4) 5 (35.7) 6 (42.9)	N = 13 3 (23.1) 8 (61.5) 2 (15.4)	N = 10 2 (20.0) 5 (50.0) 3 (30.0)	0.488
Tissue eosinophil infiltration** Absence, n (%) Mild-moderate, n (%) High, n (%)	N = 16 7 (43.7) 4 (25.0) 5 (31.2)	N = 14 9 (64.3) 1 (7.1) 4 (28.6)	N = 13 7 (53.8) 5 (38.5) 1 (7.7)	0.536	N = 14 7 (50.0) 3 (21.4) 4 (28.6)	N = 13 10 (76.9) 2 (15.4) 1 (7.7)	N = 10 7 (70.0) 2 (20.0) 1(10.0)	0.245
Granulocytes in the stroma** Absence, n (%) Mild-moderate n (%) High, n (%)	N = 16 10 (62.5) 6 (37.5) 0	N = 14 10 (71.4) 4 (28.6) 0	N = 13 9 (69.2) 3 (23.1) 1 (7.7)	0.862	N = 14 9 (64.3) 5 (35.7) 0	N = 13 11 (84.6) 2 (15.4) 0	N = 10 9 (90.0) 1 (10.0) 0	0.304
BM thickening ** Normal, n (%) Mild-moderate, n (%) High, n (%)	N = 16 0 10 (62.5) 6 (37.5)	N = 14 3 (21.4) 6 (42.9) 5 (35.7)	N = 13 2 (15.4) 8 (61.5) 3 (23.1)	0.216	N = 14 0 10 (71.4) 4 (28.6)	N = 11 1 (9.1) 9 (81.8) 1 (9.1)	N = 10 0) 6 (60.0) 4 (40.0)	0.274
Average ASM mass (%) Median [IQR] Mean (SD)	N = 16 20.0 [9.2–44.6] 25.1 (18.8)	N = 14 6.0 [0–15.0] 9.2 (11.2)	N = 13 0 [0–2.9] 4.0 (7.8)	0.001	N = 14 11.1 [1.2–17.1] 12.1 (13.3)	N = 12 4.2 [0–25.0] 12.6 (18.5)	N = 10 10.6 [2.0–20.0] 15.5 (20.9)	0.626
Distance BM-ASM μm Median [IQR] Mean (SD)	N = 12 69.6 [30.1–78.3] 67.4 (42.2)	N = 6 74.9 [23.7–195.4] 108.7 (105.6)	N = 2 46.6 46.6 (41.3)	0.667	N = 12 91.3 [48.5–129.4] 100.4 (64.9)	N = 5 54.5 [29.0–140.2] 78.5 (62.6)	N = 6 100.8 [41.3–163.0] 111.6 (82.5)	0.850

[E]	Asthma patients with relevant smoke exposure (N = 10)				Asthma patients without relevant smoke exposure (N = 20)			
	Before 1st BT	At 2^nd^ BT 1 month after 1^st^ BT	At 3^rd^ BT 1 month after 2nd BT	P value*	Before 1st BT	At 2^nd^ BT 1 month after 1st BT	At 3^rd^ BT 1 month after 2nd BT	P value*
Inflammation in the stroma** Absence, n (%) Mild-Moderate, n (%) High, n (%)	Ν = 10 1 (10.0) 6 (60.0) 3 (30.0)	Ν = 10 3 (30.0) 3 (30.0) 4 (40.0)	Ν = 7 1 (14.3) 6 (87.7) 0	0.593	Ν = 20 4 (20.0) 9 (45.0) 7 (35.0)	Ν = 17 2 (11.8) 10 (58.8) 5 (29.4)	Ν = 16 3 (18.8) 6 (37.5) 7 (43.7)	0.945
Tissue lymphocyte infiltration** Absence, n (%) Mild-moderate, n (%) High, n (%)	Ν = 10 3 (30.0) 4 (40.0) 3 (30.0)	Ν = 10 3 (30.0) 4 (40.0) 3 (30.0)	Ν = 7 1 (14.3) 6 (87.7) 0	0.915	Ν = 20 4 (20.0) 8 (40.0) 8 (40.0)	Ν = 17 2 (11.8) 11 (64.7) 4 (23.5)	Ν = 16 3 (18.8) 8 (50.0) 5 (31.2)	0.897
Tissue eosinophil infiltration** Absence, n (%) Mild-moderate, n (%) High, n (%)	Ν = 10 5 (50.0) 0 5 (50.0)	Ν = 10 8 (80.0) 0 2 (20.0)	Ν = 7 5 (71.4) 2 (28.6) 0	0.233	Ν = 20 9 (45.0) 7 (35.0) 4 (20.0)	Ν = 17 11 (64.7) 3 (17.6) 3 (17.6)	Ν = 16 9 (56.2) 5 (31.3) 2 (12.5)	0.544
Granulocytes in the stroma** Absence, n (%) Mild-moderate, n (%) High, n (%)	Ν = 10 7 (70.0) 3 (30.0) 0	Ν = 10 7 (70.0) 3 (30.0) 0	Ν = 7 5 (71.4) 2 (28.6) 0	0.981	Ν = 20 12 (60.0) 8(40.0) 0	Ν = 17 14 (82.3) 3(17.7) 0	Ν = 16 13 (81.2) 2 (12.5) 1 (6.3)	0.291
BM thickening ** Normal, n (%) Mild-moderate, n (%) High, n (%)	Ν = 10 0 7 (70.0) 3 (30.0)	Ν = 9 3 (33.3) 5 (55.6) 1 (11.1)	Ν = 7 0 7 (100.0) 0	0.162	Ν = 20 1 (5.0) 12 (60.0) 7 (35.0)	Ν = 16 2 (12.5) 10 (62.5) 4 (25.0)	Ν = 16 2 (12.5) 7 (43.8) 7 (43.8)	0.461
Average ASM mass (%) Median [IQR] Mean (SD)	Ν = 10 9.2 [1.2–22.5] 14.1 (16.8)	Ν = 10 7.5 [0.0–18.7] 13.5 (19.1)	Ν = 7 2.5 [0.0–16.2] 6.3 (8.3)	0.602	Ν = 20 17.2 [9.2–41.2] 21.5 (17.7)	Ν = 16 4.2 [0.1–10.0] 9.1 (11.7)	N = 16 2.5 [0.0–19.0] 11.1 (19.5)	0.019
Distance BM-ASM μm, Median [IQR] Mean (SD)	Ν = 8 80.7 [33.6–142.3] 100.8 (79.9)	Ν = 4 102.3 [28.7–249.8] 127.0 (121.5)	Ν = 3 87.4 [7.3–93.3] 78.4 (57.1)	0.864	Ν = 16 73.2 [48.5–94.2] 75.4 (40.4)	Ν = 7 54.5 [24.9–161.8] 76.7 (62.2)	Ν = 5 75.8 [38.4–187.6] 105.5 (92.5)	0.788

[F]	Asthma patients with T2 subtype I (N = 12)				Asthma patients with T2 subtype II (N = 9)				Asthma patients with T2 subtype III (N = 4)			
	Before 1st BT	At 2^nd^ BT 1 month after 1st BT	At 3^rd^ BT 1 month after 2^nd^ BT	P value*	Before 1st BT	At 2^nd^ BT 1 month after 1st BT	At 3^rd^ BT 1 month after 2nd BT	P value*	Before 1^st^ BT	At 2^nd^ BT 1 month after 1^st^ BT	At 3^rd^ BT 1 month after 2^nd^ BT	P value*
Inflammation in the stroma** Absence, n (%) Mild-Moderate, n (%) High, n (%)	N = 12 2 (16.7) 6 (50.0) 4 (33.3)	Ν = 11 2 (18.2) 4 (36.4) 5 (45.4)	Ν = 8 2 (25.0) 2 (25.0) 4 (50.0)	0.806	Ν = 9 1 (11.1) 5 (55.6) 3 (33.3)	Ν = 8 1 (12.5) 5 (62.5) 2 (25.0)	Ν = 7 1 (14.3) 6 (85.7) 0	0.372	Ν = 4 2 (50.0) 0 2 (50.0)	Ν = 3 1 (33.3) 0 2 (66.7)	Ν = 3 0 1 (33.3) 2 (66.7)	0.344
Tissue lymphocyte infiltration** Absence, n (%) Mild-moderate, n (%) High, n (%)	N = 12 4 (33.3) 4 (33.3) 4 (33.3)	Ν = 11 2 (18.2) 5 (45.4) 4 (36.4)	Ν = 8 2 (25.0) 3 (37.5) 3 (37.5)	0.810	Ν = 9 1 (11.1) 4 (44.4) 4 (44.4)	Ν = 8 1 (12.5) 5 (62.5) 2 (25.0)	Ν = 7 1 (14.3) 6 (85.7) 0	0.288	Ν = 4 1 (25.0) 1 (25.0) 2 (50.0)	Ν = 3 1 (33.3) 1 (33.3) 1 (33.3)	Ν = 3 0 2 (66.7) 1 (33.3)	0.450
Tissue eosinophil infiltration** Absence, n (%) Mild-moderate, n (%) High, n (%)	N = 12 3 (25.0) 4 (33.3) 5 (41.7)	Ν = 11 7 (63.6) 1 (9.1) 3 (27.3)	Ν = 8 4 (50.0) 2 (25.0) 2 (25.0)	0.316	Ν = 9 6 (66.7) 2 (22.2) 1 (11.1)	Ν = 8 7 (87.5) 1 (12.5) 0	Ν = 7 4 (57.1) 3 (42.9) 0	0.287	Ν = 4 2 (50.0) 0 2 (50.0)	Ν = 3 1 (33.3) 0 2 (66.7)	Ν = 3 1 (33.3) 2 (66.7) 0	0.787
Granulocytes in the stroma** Absence, n (%) Mild-moderate n (%) High, n (%)	N = 12 8 (66.7) 4 (33.3) 0	Ν = 11 9 (81.8) 2 (18.2) 0	Ν = 8 7 (87.5) 0 1 (12.5)	0.614	Ν = 9 6 (66.7) 3 (33.3) 0	Ν = 8 6 (75.0) 2 (25.0) 0	Ν = 7 5 (71.4) 2 (28.6) 0	0.878	Ν = 4 3 (75.0) 1 (25.0) 0	Ν = 3 2 (66.7) 1 (33.3) 0	Ν = 3 2 (66.7) 1 (33.3) 0	0.945
BM thickening ** Normal, n (%) Mild-moderate, n (%) High, n (%)	N = 12 0 10 (83.3) 2 (16.7)	Ν = 9 2 (22.2) 5 (55.6) 2 (22.2)	Ν = 8 1 (12.5) 5 (62.5) 2 (25.0)	0.786	Ν = 9 1 (11.1) 6 (66.7) 2 (22.2)	Ν = 8 1 (12.5) 6 (75.0) 1 (12.5)	Ν = 7 1 (14.3) 5 (71.4) 1 (14.3)	0.872	Ν = 4 0 1 (25.0) 3 (75.0)	Ν = 3 1 (33.3) 0 2 (66.7)	Ν = 3 0 1 (33.3) 2 (66.7)	0.350
Average ASM mass (%) Median [IQR] Mean (SD)	N = 12 15.1 [7.8–26.4] 17.1 (14.6)	Ν = 10 4.1 [0.8–19.2] 10.0 (12.8)	Ν = 8 9.8 [2.2–18.9] 10.6 (10.0)	0.290	N = 9 20.0 [8.1–40.0] 24.1 (20.7)	Ν = 8 5.3 [-0.5–16.0] 7.7 (9.8)	Ν = 7 0 [-3.1–9.2] 3.1 (6.6)	0.049	N = 4 26.9 [-7.1–65.1] 29.0 (22.7)	Ν = 3 10.0 [-10.6–27.3] 8.3 (7.6)	Ν = 3 0 [-2.7–4.4] 0.8 (1.4)	0.082
Distance BM-ASM μm Median [IQR] Mean (SD)	Ν = 10 84.8 [51.0–153.8] 102.4 (71.8)	Ν = 7 93.3 [43.9–146.8] 95.3 (55.6)	Ν = 3 75.8 [-33.2–153.5] 60.2 (37.6)	0.639	Ν = 5 71.5 [14.3–132.5] 73.4 (47.6)	Ν = 1 19.9	Ν = 1 114.3	–	Ν = 4 65.7 [26.6–93.7] 60.1 (21.1)	Ν = 1 296.0	Ν = 0	–

T2 subtype I: Patients with only one T2 positive marker (allergy, or atopy, or eosinophils ≥ 300/μL)

T2 subtype II: Patients with any two of T2 positive markers

T2 subtype III: Patients with any three of T2 positive markers

^ǂ^Cannot be determined, only 1 observation in two time points

^*^Comparisons between values before and after BT. For categorical values, the p value was calculated using mixed multinomial (ordinal) logistic regression models, where the factor patient was included as a random effect. For continuous values, the p value was calculated using rank mixed linear regression models, where the factor patient was included as a random effect

^**^Qualitative evaluation, 0–3 scale: 0 to < 0.5 = absence/normal, 0.5 to < 1.50 = mild-moderate, 1.5–3 = high

*BT* bronchial thermoplasty, *BM* basement membrane, *ASM* airway smooth muscle, *IQR* inter quartile range, *SD* standard deviation

Across distinct endotypes/phenotypes, patients presented a significant decrease in ASM mass after bronchial thermoplasty were the ones with T2 high asthma endotype, as patients with high blood eosinophils (p = 0.009), atopy (p = 0.012), allergy (p = 0.001) and without smoke exposure (p = 0.019) (Table 2B–E).

When patients with T2 positive markers (allergy, atopy, high blood eosinophils) were stratified in subtypes according to the number of T2 positive markers, a significant decrease in ASM mass after bronchial thermoplasty (p = 0.049), was observed in patients with T2 subtype II, that is patients with any two of the T2 positive markers (Table 2F).

Using linear regression models, we observed that there was no significant association between blood eosinophils and tissue eosinophilic infiltration before bronchial thermoplasty (p = 0.474), after the first (p = 0.487) and after the second bronchial thermoplasty (p = 0.135) (Additional file 3: Table S2). Furthermore, there was no difference in the effect of bronchial thermoplasty on any of the histological parameters evaluated between patients with (n = 16) and without (n = 14) tissue eosinophilic infiltration (Additional file 4: Table S3).

### Thermoplasty stimulates proliferation of epithelium

**Fig. 3:**
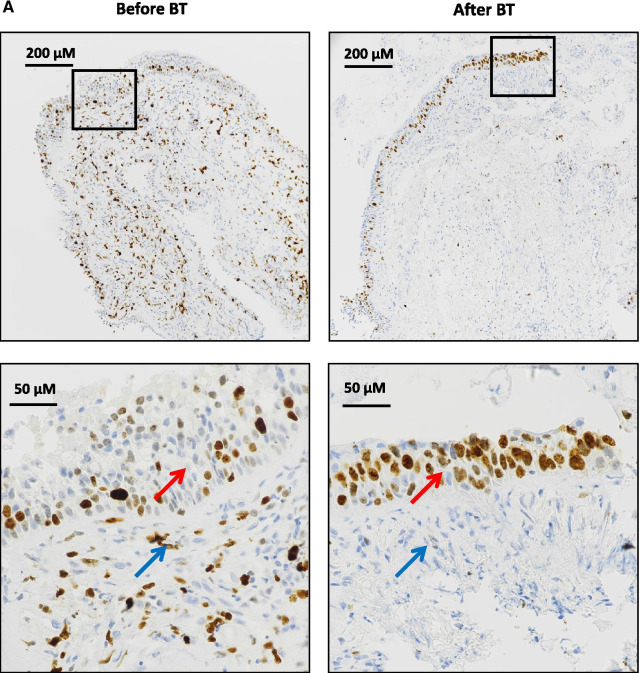

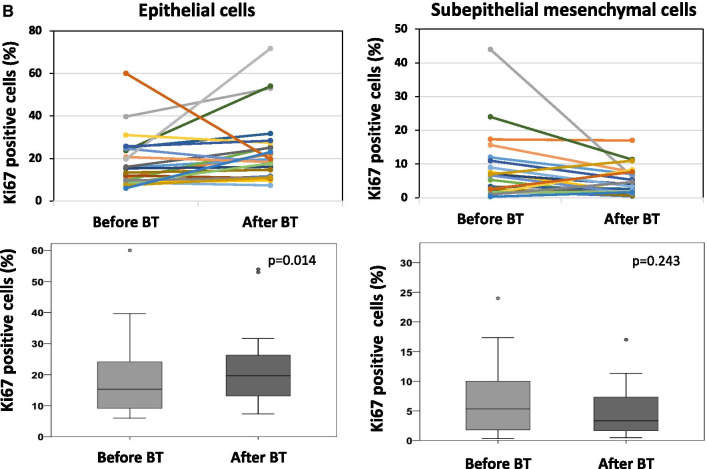
Proliferative epithelial cells and subepithelial mesenchymal cells in endobronchial biopsies (EBB) obtained before and after BT was assessed by staining with antibodies for Ki67, a nuclear protein that is expressed only in proliferating cells. **A** Representative microphotographs showing epithelial cells (red arrows) and subepithelial mesenchymal cells (blue arrows) stained positive for Ki67 (brown color). Photographs were captured by Olympus IX83 microscope, using a DS-Ri2 color imaging camera. Right panels show enlargement of the areas in black boxes. **B** Counting of proliferative cells that stained positive for Ki67 was performed in 3 randomly selected areas on each EBB under the × 200 magnification of the microscope (Nikon Eclipse Ti2 inverted microscope system). Results were expressed as number of Ki67 positive cells per 100 nuclei (%). Upper panels show paired data (from the same patient) before and after BT. In lower panels, horizontal lines in box plots represent median values. Comparisons were made by the Wilcoxon signed rank test. *BT* bronchial thermoplasty

Cells undergoing active proliferation were assessed in EBB obtained before and 2 months after the 3rd bronchial thermoplasty by immunostaining for Ki67 protein (Fig. 3A). Quantitation of Ki67 + cells revealed that after bronchial thermoplasty, the number of proliferative (Ki67 +) epithelial cells was significantly increased (p = 0.014) (Fig. 3B, Table 3).

**Table 3:** Expression of Ki67 in endobronchial biopsies from patients with different asthma endotypes/phenotypes before and after bronchial thermoplasty

Asthma endotypes/phenotypes	Expression of Ki67 (%)^#^	Epithelial cells			Sub-epithelial mesenchymal cells		
		Before BT	After BT	P Value*	Before BT	After BT	P Value*
eos ≥ 300/μL (N = 7)	Mean ± SD	22.9 ± 17.4	24.1 ± 15.1	0.617	6.1 ± 8.1	4.3 ± 3.9	0.900
	Median (IQR)	16.0 (12.0–25.0)	19.8 (11.8–31.8)		2.8 (2.0–7.0)	2.8 (1.3–7.8)	
eos < 300/μL (N = 16)	Mean ± SD	16.3 ± 9.8	24.0 ± 17.2	0.016	8.4 ± 10.6	4.8 ± 4.6	0.283
	Median (IQR)	12.2 (8.8–23.2)	19.8 (11.0–27.3)		6.2 (1.5–10.0)	3.3 (1.8–7.0)	
Atopy (N = 16)	Mean ± SD	21.0 ± 14.1	24.8 ± 13.2	0.144	8.9 ± 11.4	5.7 ± 4.8	0.540
	Median (IQR)	18.8 (9.7–25.3)	22.0 (17.7–28.3)		4.5 (1.8–11.5)	5.3 (1.7–8.3)	
No Atopy (N = 7)	Mean ± SD	12.1 ± 4.2	22.2 ± 22.6	0.098	4.8 ± 3.2	2.4 ± 1.6	0.173
	Median (IQR)	9.7 (8.7–15.3)	14.7 (9.7–25.3)		5.3 (1.3–7.7)	2.0 (0.7–4.0)	
Allergy (N = 12)	Mean ± SD	19.2 ± 14.6	21.0 ± 12.6	0.243	8.0 ± 6.8	6.0 ± 5.2	0.216
	Median (IQR)	15.8 (8.5–24.2)	18.0 (11.8–25.0)		6.8 (2.3–10.0)	4 (1.8–11.0)	
No allergy (N = 11)	Mean ± SD	17.3 ± 10.6	27.1 ± 19.3	0.028	7.3 ± 12.6	3.4 ± 2.7	0.796
	Median (IQR)	13.3 (9.8–25.0)	23.0 (11.0–31.8)		2.8 (1.3–7.8)	2.0 (1.3–6.3)	
Smoke exposure (N = 9)	Mean ± SD	19.6 ± 17.6	24.1 ± 19.3	0.234	3.9 ± 4.0	5.1 ± 3.3	0.155
	Median (IQR)	9.8 (8.3–25.8)	19.8 (11.0–27.3)		1.8 (1.3–7.0)	4.8 (2.0–7.8)	
No smoke exposure (N = 14)	Mean ± SD	17.5 ± 8.7	23.9 ± 14.6	0.034	10.1 ± 11.7	4.4 ± 4.9	0.001
	Median (IQR)	15.5 (11.0–23.8)	19.8 (14.8–25.3)		6.2 (2.8–12.0)	2.8 (1.3–6.3)	
All patients (N = 23)	Mean ± SD	18.3 ± 12.6	24.0 ± 16.2	0.014	7.7 ± 9.8	4.7 ± 4.3	**0.243**
	Median (IQR)	15.3 (12.9–23.7)	19.7 (16.8–31.2)		5.3 (3.4–11.9)	3.0 (2.8–6.6)	

^**#**^Ki67 positive cells per 100 nuclei

*The p value for comparisons between values before and after BT was calculated using ranked mixed linear regression models, where the factor patient was included as a random effect

Across distinct asthma endotypes/phenotypes (Table 3), proliferative epithelial cells were significantly increased after bronchial thermoplasty in asthma patients with low blood eosinophils (p = 0.016), without allergy (p = 0.028) and no smoke exposure (p = 0.034). The number of proliferative subepithelial mesenchymal cells was significantly decreased only in patients without smoke exposure (p = 0.001).

### Thermoplasty increases glucocorticoid receptor expression

**Fig. 4:**
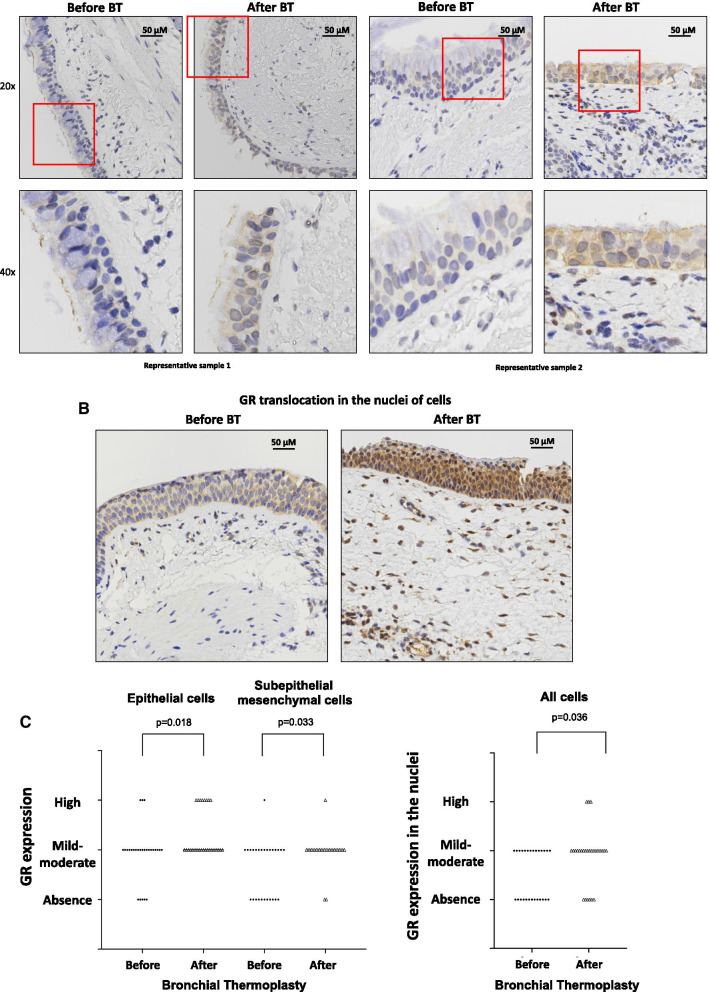
Expression of glucocorticoid receptor (GR) in endobronchial biopsies obtained from severe asthma patients using a specific monoclonal antibody. **A** Representative microphotographs. Lower panels show enlargement of the areas in red boxes. **B** Representative microphotographs showing increased localization of GR in the nuclei after BT. **C** expression score in epithelial cells, subepithelial mesenchymal cells and in the nuclei of all cells from patients with severe asthma before and after thermoplasty. The p value was calculated using mixed multinomial (ordinal) logistic regression models, where the factor patient was included as a random effect. *BT* bronchial thermoplasty

Immunohistochemistry in EBB before and after bronchial thermoplasty revealed that immunoreactivity for GR was observed in both epithelial cells and subepithelial mesenchymal cells both in the cytoplasm and in the nuclei (Figs. 4A, B). After bronchial thermoplasty, there was a significant increase in the expression of GR in epithelial cells (p = 0.018) and in subepithelial mesenchymal cells (p = 0.033) (Fig. 4C). Furthermore, the accumulation of GR in the nuclei of all cells was significantly increased (p = 0.036) after bronchial thermoplasty, indicating its activation (Fig. 4C).

Across distinct asthma endotypes/phenotypes the group of patients with low blood eosinophils (< 300/μl), presented a significant increase of GR expression in epithelial cells (p = 0.044) and accumulation of GR in the nuclei (p = 0.044) after bronchial thermoplasty (Table 4).

**Table 4:** Expression of glucocorticoid receptor in endobronchial biopsies from patients with different asthma endotypes/phenotypes before and after bronchial thermoplasty

Asthma endotypes-phenotypes	Expression of GR	Epithelial cells			Sub-epithelial mesenchymal cells			Nuclei of all cells		
		Before BT	After BT	P value*	Before BT	After BT	P value*	Before BT	After BT	P value*
eos ≥ 300/μL (N = 7)	Absence, n (%) Mild-Moderate, n (%) High, n (%)	3 (42.9) 3 (42.9) 1 (14.3)	0 6 (66.7) 1 (14.3)	0.205	4 (57.1) 2 (28.6) 1 (14.3)	1 (14.3) 5 (71.4) 1 (14.3)	0.222	5 (71.4) 2 (28.6) 0	4 (57.1) 2 (28.6) 1 (14.3)	0.497
eos < 300/μL (N = 19)	Absence, n (%) Mild-Moderate, n (%) High, n (%)	2 (27.8) 15 (55.5) 2 (16.7)	0 12 (68.4) 7 (31.6)	0.044	6 (44.4) 13 (50.0) 0	1 (5.3) 18 (94.7) 0	0.071	7 (44.4) 12 (55.5) 0	2 (10.5) 15 (78.9) 2 (10.5)	0.044
Atopy (N = 16)	Absence, n (%) Mild-Moderate, n (%) High, n (%)	4 (25.0) 10 (62.5) 2 (12.5)	0 12 (75.0) 4 (25.0)	0.083	8 (50.0) 7 (43.7) 1 (6.3)	2 (12.5) 13 (81.2) 1 (6.3)	0.059	8 (50.0) 8 (50.0) 0	4 (25.0) 10 (62.5) 2 (12.5)	0.097
No Atopy (N = 10)	Absence, n (%) Mild-Moderate, n (%) High, n (%)	1 (10.0) 8 (80.0) 1 (10.0)	0 7 (70.0) 3 (30.0)	0.124	2 (20.0) 8 (80.0) 0	0 10 (100.0) 0	0.975	4 (40.0) 6 (60.0) 0	2 (20.0) 7 (70.0) 1 (10.0)	0.253
Allergy (N = 14)	Absence, n (%) Mild-Moderate, n (%) High, n (%)	4 (23.1) 8 (61.5) 2 (15.4)	0 11(76.9) 3 (23.1)	0.124	6 (38.5) 7 (53.8) 1 (7.7)	2 (15.4) 11 (76.9) 1 (7.7)	0.169	7 (46.1) 7 (53.9) 0	5 (38.5) 7 (46.1) 2 (15.4)	0.284
No allergy (N = 12)	Absence, n (%) Mild-Moderate, n (%) High, n (%)	1 (8.3) 10 (83.3) 1 (8.3)	0 7 (68.4) 5 (31.6)	0.078	4 (33.3) 8 (66.7) 0	0 12 (100.0) 0	0.977	5 (41.7) 7 (58.3) 0	1 (8.3) 10 (83.3) 1 (8.3)	0.078
Smoke exposure (N = 9)	Absence, n (%) Mild-Moderate, n (%) High, n (%)	0 9 (100.0) 0	0 6 (66.7) 3 (33.3)	0.970	3 (33.3) 6 (66.7) 0	1 (11.1) 7 (77.8) 1 (11.1)	0.206	5 (55.6) 4 (44.4) 0	2 (22.2) 6 (66.7) 1 (11.1)	0.140
No smoke exposure (N = 17)	Absence, n (%) Mild-Moderate, n (%) High, n (%)	5 (29.4) 9 (52.9) 3 (17.6)	0 12 (70.6) 5 (29.4)	0.073	7 (41.2) 9 (52.9) 1 (5.9)	1 (5.9) 16 (94.1) 0	0.072	7 (41.2) 10 (58.8) 0	4 (23.5) 11 (64.7) 2 (11.8)	0.162

*Comparisons between the groups were performed using a multinomial logistic regression model

*BT* bronchial thermoplasty, *GR* glucocorticoid receptor

### Thermoplasty alters HSPs expression

**Fig. 5:**
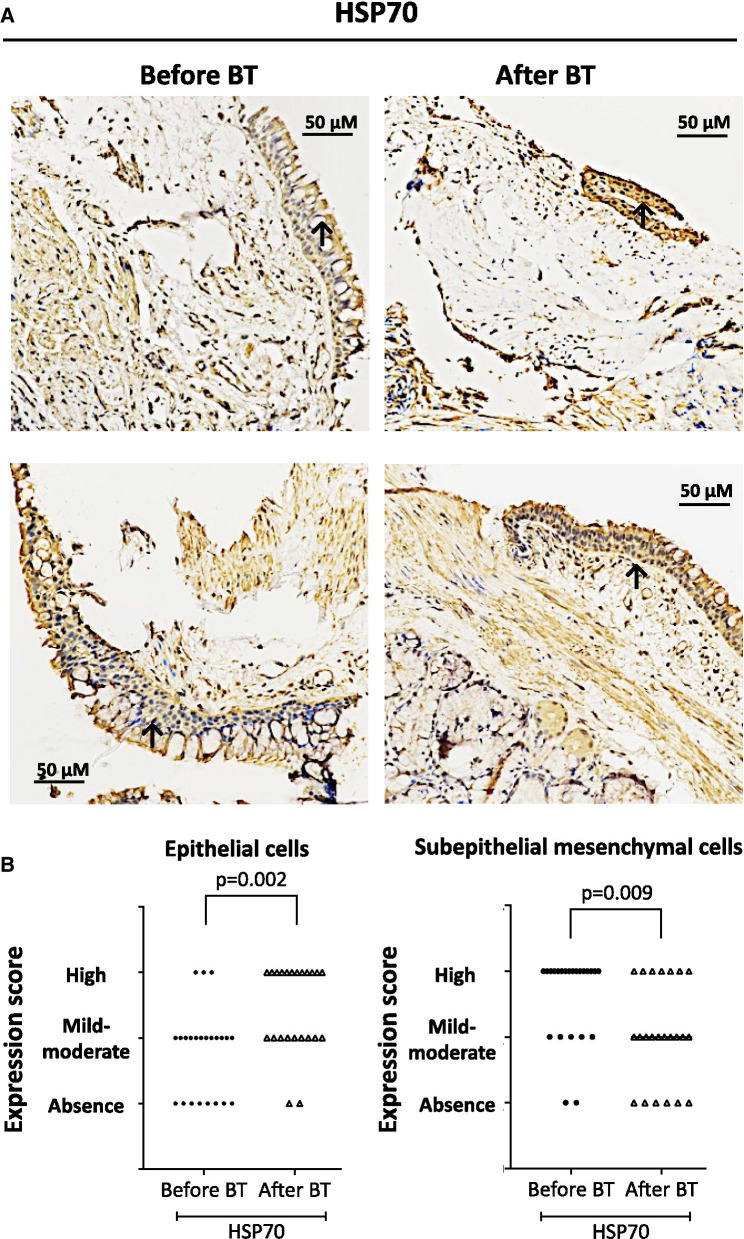
Expression of heat shock protein 70 (HSP70) in endobronchial biopsies obtained from severe asthma patients using a specific monoclonal antibody. **A** Representative microphotographs showing the expression of HSP70 before and after bronchial thermoplasty (BT). **B** HSP70 expression score in epithelial cells and subepithelial mesenchymal cells from 30 asthma patients before and after BT. The p value was calculated using mixed multinomial (ordinal) logistic regression models, where the factor patient was included as a random effect

**Fig. 6:**
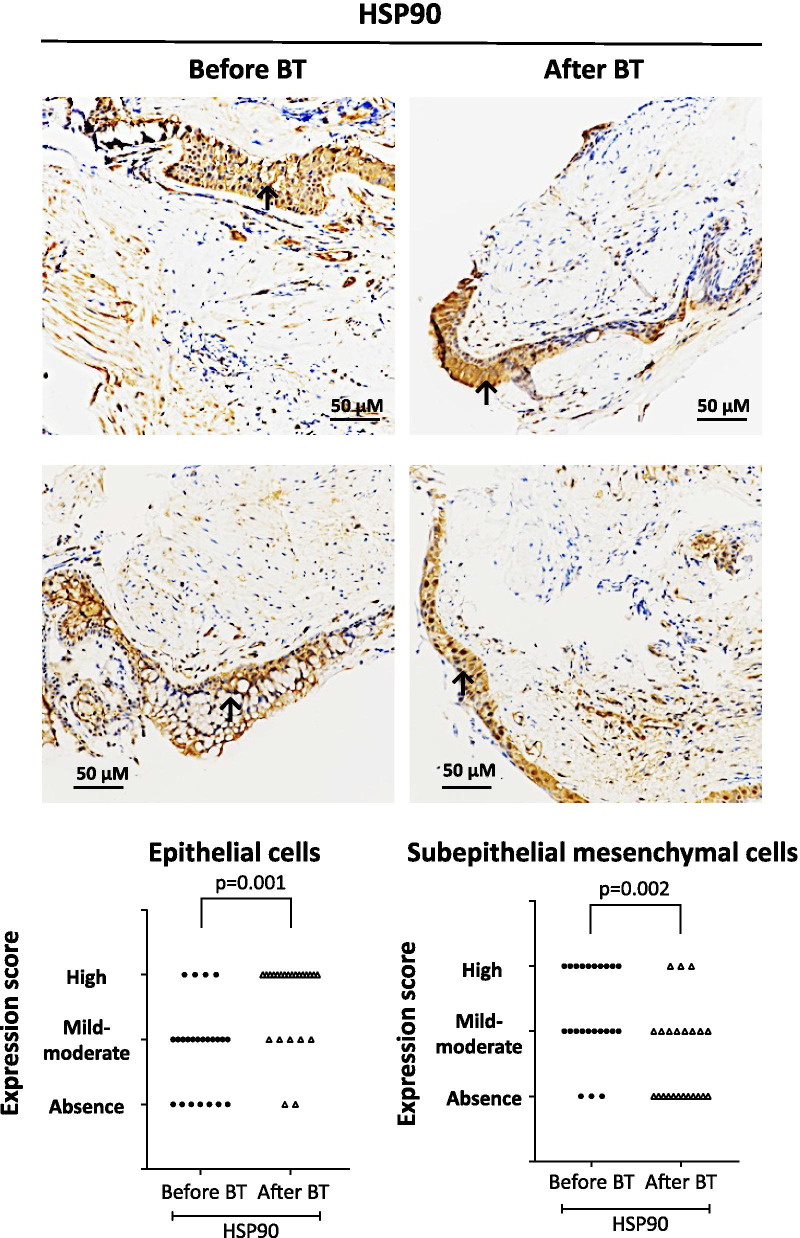
Expression of heat shock protein 90 (HSP90) in endobronchial biopsies obtained from severe asthma patients using a specific monoclonal antibody. **A** Representative microphotographs showing the expression of HSP90 before and after bronchial thermoplasty (BT). **B** HSP90 expression score in epithelial cells and subepithelial mesenchymal cells from 30 asthma patients before and after BT. The p value was calculated using mixed multinomial (ordinal) logistic regression models, where the factor patient was included as a random effect

Endobronchial biopsies obtained from severe asthma patients before and after bronchial thermoplasty stained positively for HSP70 and HSP90 antigens (Figs. 5A, 6A). Quantitation of HSP70 + and HSP90 + cells revealed that tissue expression of HSP70 and HSP90 was significantly increased in epithelial cells after bronchial thermoplasty (p = 0.002 and p = 0.001, respectively) (Figs. 5B, 6B). In subepithelial mesenchymal cells, there was a significant decrease in the expression of HSP70 (p = 0.009) and HSP90 (p = 0.002) after bronchial thermoplasty (Figs. 5B, 6B).

Across distinct asthma endotypes/phenotypes, comparison between different groups suggested differences in the expression of HSP70 after bronchial thermoplasty in patients with atopy, in epithelial cells (increased expression, p = 0.011 vs p = 0.117 for patients without atopy), and subepithelial mesenchymal cells (decreased expression, p = 0.018 vs p = 0.063 for patients without atopy) (Table 5). However, when comparisons were made between the groups only smoke exposure was associated with significant differences in HSP70 expression in epithelial cells and subepithelial mesenchymal cells (p = 0.045 and p = 0.003, respectively, for comparisons between patients with and without smoke exposure).

**Table 5:** Expression of HSP70 in endobronchial biopsies from patients with different asthma endotypes/phenotypes before and after bronchial thermoplasty

Asthma endotypes-phenotypes	Expression of HSP70	Epithelial cells			P Value^a^	Subepithelial mesenchymal cells			P Value^a^
		Before BT	After BT	P Value*		Before BT	After BT	P Value*	
eos ≥ 300/μL (N = 7)	Absence, n (%) Mild-Moderate, n (%) High, n (%)	3 (42.8) 3 (42.8) 1(14.3)	0 2 (28.6) 5 (71.4)	0.047	0.287	0 0 7 (100)	2 (28.6) 4 (57.1) 1 (14.3)	0.973	0.975
eos < 300/μL (N = 16)	Absence, n (%) Mild-Moderate, n (%) High, n (%)	5 (31.2) 9 (56.2) 2 (12.5)	1 (6.7) 7 (46.7) 7 (46.7)	0.029		0 6 (37.5) 10 (62.5)	3 (20.0) 7 (46.7) 5 (33.3)	0.063	
Atopy (N = 16)	Absence, n (%) Mild-Moderate, n (%) High, n (%)	7 (43.7) 7 (43.7) 2 (12.5)	1 (6.7) 6 (40.0) 8 (53.3)	0.011	0.555	0 5 (31.2) 11 (68.8)	3 (20.0) 8 (53.3) 4 (26.7)	0.018	0.446
No atopy (N = 7)	Absence, n (%) Mild-Moderate, n (%) High, n (%)	1 (14.3) 5 (71.4) 1 (14.3)	0 3 (42.8) 4 (57.1)	0.117		0 1 (14.3) 6 (85.7)	2 (28.6) 3 (42.8) 2 (28.6)	0.063	
Allergy (N = 12)	Absence, n (%) Mild-Moderate, n (%) High, n (%)	6 (50.0) 5 (41.7) 1 (8.3)	1 (9.1) 5 (45.5) 5 (45.5)	0.028	0.670	0 3 (25.0) 9 (75.0)	1 (9.1) 6 (54.5) 4 (36.4)	0.073	0.324
No allergy (N = 11)	Absence, n (%) Mild-Moderate, n (%) High, n (%)	2 (18.2) 7 (63.6) 2 (18.2)	0 4 (36.4) 7 (63.6)	0.037		0 3 (27.3) 8 (72.7)	4 (36.4) 5 (45.5) 2 (18.1)	0.015	
Smoke exposure (N = 9)	Absence, n (%) Mild-Moderate, n (%) High, n (%)	1 (11.1) 6 (66.7) 2 (22.2)	1 (11.1) 4 (36.4) 4 (36.4)	0.429	0.045	0 5 (55.6) 4 (44.4)	1 (11.1) 3 (33.3) 5 (55.6)	0.840	0.003
No smoke exposure (N = 14)	Absence, n (%) Mild-Moderate, n (%) High, n (%)	7 (50.0) 6 (42.9) 1 (7.1)	0 5 (38.5) 8 (61.5)	0.006		0 1 (7.1) 13 (92.9)	4 (30.8) 8 (61.5) 1 (7.7)	0.020	

*Comparisons between the groups were performed using a multinomial logistic regression model

^a^Difference in differences between groups were performed by introducing the interaction of time and group classification in a mixed logistic regression

*BT* bronchial thermoplasty, *HSP* heat shock protein

Similarly, after bronchial thermoplasty, a significant increase was observed in the expression of HSP90 in epithelial cells in patients with low blood eosinophils (p = 0.006 vs. p = 0.976 for patients with high blood eosinophils) and atopy (p = 0.008 vs. p = 0.570 for patients without atopy) and a significant decrease in the expression of HSP90 in subepithelial mesenchymal cells in patients with atopy (p = 0.008 vs. p = 0.093 for patients without atopy) (Table 6). However, when comparisons were made between the groups only smoke exposure was associated with significant differences in HSP90 expression in subepithelial mesenchymal cells (p = 0.003, for comparisons between patients with and without smoke exposure).

**Table 6:** Expression of HSP90 in endobronchial biopsies from patients with different asthma endotypes/phenotypes before and after bronchial thermoplasty

Asthma endotypes-phenotypes	Expression of HSP90	Epithelial cells			P Value^a^	Subepithelial mesenchymal cells			P Value^a^
		Before BT	After BT	P value*		Before BT	After BT	P value*	
eos ≥ 300/μL (N = 6)	Absence, n (%) Mild-Moderate, n (%) High, n (%)	1 (16.7) 5 (83.3) 0	0 2 (33.3) 4 (66.7)	0.976	0.518	0 2 (33.3) 4 (66.7)	3 (50.0) 2 (33.3) 1 (16.7)	0.039	0.215
eos < 300/μL (N = 16)	Absence, n (%) Mild-Moderate, n (%) High, n (%)	6 (37.5) 7 (43.8) 3 (18.7)	1 (6.7) 3 (20.0) 11 (73.3)	0.006		2 (12.5) 9 (56.2) 5 (31.3)	8 (53.3) 6 (40.0) 1 (6.7)	0.018	
Atopy (N = 15)	Absence, n (%) Mild-Moderate, n (%) High, n (%)	4 (26.7) 10 (66.7) 1 (6.7)	1 (7.1) 4 (28.6) 9 (64.3)	0.008	0.350	1 (6.7) 7 (46.7) 7 (46.7)	7 (50.0) 5 (35.7) 2 (14.3)	0.008	0.699
No atopy (N = 7)	Absence, n (%) Mild-Moderate, n (%) High, n (%)	3 (42.8) 2 (28.6) 2 (28.6)	0 1 (14.3) 6 (85.7)	0.570		1 (14.3) 4 (57.1) 2 (28.6)	4 (57.1) 3 (42.9) 0	0.093	
Allergy (N = 12)	Absence, n (%) Mild-Moderate, n (%) High, n (%)	3 (18.2) 8 (72.7) 1 (9.1)	1 (10.0) 3 (20.0) 7 (70.0)	0.025	0.599	1 (9.1) 6 (45.5) 5 (45.5)	5 (40.0) 4 (40.0) 2 (20.0)	0.073	0.350
No allergy (N = 10)	Absence, n (%) Mild-Moderate, n (%) High, n (%)	4 (40.0) 4 (40.0) 2 (20.0)	0 2 (20.0) 8 (80.0)	0.015		1 (10.0) 5 (50.0) 4 (40.0)	6 (60.0) 4 (40.0) 0	0.013	
Smoke exposure (N = 9)	Absence, n (%) Mild-Moderate, n (%) High, n (%)	2 (22.2) 4 (44.4) 3 (33.3)	1 (11.1) 3 (33.3) 5 (55.6)	0.343	0.059	2 (22.2) 5 (55.6) 2 (22.2)	2 (22.2) 5 (55.6) 2 (22.2)	0.100	0.003
No smoke exposure (N = 13)	Absence, n (%) Mild-Moderate, n (%) High, n (%)	5 (38.5) 8 (61.5) 0	0 2 (16.7) 10 (83.3)	0.973		0 6 (46.2) 7 (53.8)	9 (75.0) 3 (25.0) 0	0.955	

*Comparisons between the groups were performed using a multinomial logistic regression model

^a^Difference in differences between groups were performed by introducing the interaction of time and group classification in a mixed logistic regression

*BT* bronchial thermoplasty, *HSP* heat shock protein

## Discussion

In the present study, we evaluated the effect of bronchial thermoplasty on histological parameters in 30 well-characterized patients with severe asthma, including various endotypes/phenotypes. The main novel finding of our study is that depending on the asthma endotype/phenotype, bronchial thermoplasty results in distinct histopathological changes including decreased bronchial ASM mass, regeneration of bronchial epithelial cells, increased expression and activation of GR in the airways and increased expression of HSPs in bronchial epithelium. Thus, bronchial thermoplasty targets alternative molecular pathways associated with asthma endotypes/phenotypes.

An important goal of asthma management strategies is to improve patients’ daily activities and to control asthma symptoms. In line with previous studies, [3, 4, 6, 18] bronchial thermoplasty did not alter postbronchodilator lung function parameters, however, significantly improved asthma control, as assessed by daily symptoms using ACT. This has been a validated method to reflect asthma control [19–21]. It has been suggested that a difference of 3 points for the ACT is a clinically meaningful change in asthma control in an individual patient overtime [22]. In the present study, patients achieved a difference of 3.8 points for ACT total score after bronchial thermoplasty, indicating a relevant improvement in asthma symptoms. Changes in ACT total score after bronchial thermoplasty were similar across distinct endotypes/phenotypes implying that the procedure has a beneficial effect on patients’ asthma symptoms control, irrespective their asthma endotype/phenotype.

Histopathologic examination of bronchial biopsy specimens collected at each sequential bronchial thermoplasty showed a significant reduction of ASM mass by 68.4% after the first bronchial thermoplasty and by 81.2% after the second bronchial thermoplasty, confirming the results obtained in previous studies [6, 23]. The decrease of ASM after bronchial thermoplasty was significant for patients with high blood eosinophils, atopy, allergy, and no smoke exposure, implying that patients with T2 high asthma may profit more from the procedure.

In the present study, the number of proliferating, (Ki67 +) epithelial cells, was significantly increased in asthma patients after bronchial thermoplasty, an observation that is in line with the rapid reconstruction of the epithelium after bronchial thermoplasty shown in previous studies [6, 24, 25]. Across distinct asthma endotypes/phenotypes, proliferating epithelial cells were significantly increased after bronchial thermoplasty in patients with low blood eosinophils, in patients without allergy and in patients without smoke exposure, indicating that the procedure activates similar mechanisms for epithelial cell repair in these groups of patients.

On the contrary, after bronchial thermoplasty, the number of Ki67 + subepithelial mesenchymal cells was numerically decreased, however, no significantly. This is not surprising, since the number of actively proliferating ASMC is low in subjects with asthma of varying severity, including subjects with severe asthma as those included in the present study [26]. Moreover, this observation is in line with the reduction of ASM mass observed after bronchial thermoplasty. Across distinct asthma endotypes/phenotypes, proliferating subepithelial mesenchymal cells were significantly decreased after bronchial thermoplasty only in patients without smoke exposure. However, patients without smoke exposure were presented with higher amount of ASM before bronchial thermoplasty [median (IQR): 17.2 (9.2–41.2) versus 9.2 (1.2–22.5) for patients with smoke exposure].

Although ASM mass was significantly decreased after bronchial thermoplasty there was no change in BM thickness or in the distance between BM and ASMC before and after bronchial thermoplasty in asthma patients of any endotype/phenotype. It has been suggested that besides a direct beneficial effect of bronchial thermoplasty in reducing ASMC, the therapeutic outcome of this procedure goes beyond the treated areas and influences more peripheral airways, resulting in a reduction of small airway narrowing and air-trapping [27, 28]. Therefore, it is tempting to hypothesize that the lack of significant differences in BM thickening and in the distance between BM and ASMC before and after bronchial thermoplasty, may reflect a moderate local modification of these parameters, however, distributed to distal areas of the bronchial tree and resulting in an overall significant improvement in airway remodeling and patients’ symptoms.

We have previously shown that a potential mechanism for the effect of bronchial thermoplasty on airway remodeling involves the impediment of epithelium-derived HSP60 secretion and PRMT1 in fibroblasts [11]. HSPs belong to a family of proteins that are induced in all cells when there is a threat of the cellular environment, such as inflammation. Based on our previous findings that HSPs mediate cellular pathways associated with airway remodeling, [12] we assessed tissue expression of HSP70 and HSP90 in EEB obtained before and after bronchial thermoplasty. HSP70 is a molecular chaperon and has been associated with a possible role in antigen processing and presentation [29]. Thus, HSP70 may enhance a protective immune response or a harmful allergic response. It is still unclear if overexpression of HSP70 in asthma represents increased stress or an autoprotective mechanism. In vivo experiments have shown that HSP70 protects against pulmonary inflammation and sepsis [30] and decrease mortality in an animal model of acute lung injury [31]. Furthermore, HSP70 has been reported to prevent cell apoptosis [32, 33]. The above evidence suggests that the increased expression of HSP70 in epithelial cells after bronchial thermoplasty shown in the present study, inhibits epithelial cell apoptosis and thereby helps to rebuild a functional epithelium. This hypothesis is confirmed by the increased number of proliferating (Ki67 +) epithelial cells that we observed in EBB after bronchial thermoplasty. On the other hand, HSP70 expression in subepithelial mesenchymal cells is decreased after bronchial thermoplasty and this may lead to increased apoptosis and a lower number of proliferative (Ki67 +) subepithelial mesenchymal cells that we report in the present study.

HSP90 is the most abundant HSP in eukaryotic cells with two clearly signed roles both of which are important for the cells to cope with environmental changes such as tissue injury and heat. One of these roles is to act as an extracellular tissue-repairing factor as it has been shown that HSP90 promotes re-epithelialization and cell motility [34, 35]. Thus, the observation that after bronchial thermoplasty there is an increased expression of HSP90 in epithelial cells and a decreased expression in subepithelial mesenchymal cells indicates a possible cell-type specific mechanism of epithelial cell regeneration after bronchial thermoplasty that explains the beneficial outcomes of the procedure [12].

The second role of HSP90 is to act as an intracellular chaperone that forms heterocomplexes with GR, a process that also requires HSP70 [36, 37]. A critical role of the HSPs is to facilitate the folding of the hormone-binding domain of the receptor into a high-affinity steroid binding conformation. The main function of the GR/HSP complex is to keep the receptor in an inactive, yet potential ligand-active state. Therefore, the increased expression of HSP70 and HSP90 in epithelial cells after bronchial thermoplasty reported in the present study, facilitates GR function by increasing its binding to glucocorticoids, resulting in improved pharmacological action in asthma patients across all endotypes/phenotypes. These findings also provide a reasonable explanation for the clinically documented reduced need for corticosteroids after bronchial thermoplasty [38, 39].

Another novel finding of our study that also justifies the improved response of asthma patients to glucocorticoid treatment after bronchial thermoplasty, is the increased expression of GR in epithelial cells and in subepithelial mesenchymal cells after the procedure. In a model of chronic injury of mucociliated human bronchial epithelial cells, it has been shown that glucocorticoids increase repair potential [40]. In bronchial tissue sections of asthma patients, the expression of several proproliferative proteins in the epithelium correlated with the dosage of therapeutic glucocorticoids [41]. During lung maturation the GR is the key regulator of mesenchymal cell differentiation [42]. Furthermore, it has been shown that glucocorticoids inhibit the proliferation of ASMC, through the activation of the GR [43, 44]. However, the anti-proliferative effect of glucocorticoids depends on the presence of various co-factors such as C/EBP-alpha or insulin growth factor binding protein-1 [45, 46]. Thus, the increased expression of GR in epithelial cells and in subepithelial mesenchymal cells after bronchial thermoplasty, that we report in this study, may be a key regulator of the increased proliferation of epithelial cells and the decreased proliferation of subepithelial mesenchymal cells after the procedure. Overall, the observed increased expression of the GR and HSP90 in epithelial cells and subepithelial mesenchymal cells may explain the lasting beneficial effect of bronchial thermoplasty on the airway wall structure, as well as the reduced necessity of inhaled glucocorticoids after bronchial thermoplasty.

A limitation of our study is that it is not controlled as healthy individuals could not be subjected to such an invasive procedure and sham bronchial thermoplasty would also raise ethical issues. However, a major strength of our study is the assessment of biopsies that were obtained longitudinally after sequential bronchial thermoplasties from the same location allowing precise comparisons.

## Conclusions

Our study implies that bronchial thermoplasty is associated with epithelial cell regeneration, decrease of ASM, increased expression and activation of GR, and increased expression of HSPs in the airways of asthma patients. Histopathological effects appear to be distinct in different endotypes/phenotypes indicating that the beneficial effects of bronchial thermoplasty are achieved by diverse molecular targets associated with asthma endotypes/phenotypes.

## Supplementary Information

**Additional file 1: Figure S1.** Representative microphotographs of endobronchial biopsies obtained from asthma patients before and after thermoplasty, at different magnifications (X100-X400).

**Additional file 2: Table S1.** Lung function parameters in patients with severe asthma after bronchial thermoplasty.

**Additional file 3: Table S2.** Association between blood eosinophils and tissue eosinophilic infiltration before BT using generalized linear models.

**Additional file 4: Table S3.** Histopathological evaluation of endobronchial biopsies before and after bronchial thermoplasty.

## Data Availability

The datasets used and/or analysed during the current study are available from the corresponding author on reasonable request.

## Abbreviations

ACT: Asthma Control Test
ASM: Airway smooth muscle
ASMC: Airway smooth muscle cells
BM: Basement membrane
EBB: Endobronchial biopsies
GR: Glucocorticoid receptor
HSP70: Heat shock protein 70
HSP90: Heat shock protein 90
PY: Pack-years
