# Supplementary material for: Bronchial thermoplasty in asthma: an exploratory histopathological evaluation in distinct asthma endotypes/phenotypes
Source: Respir Res. 2021 Jun 28;22:186. doi: 10.1186/s12931-021-01774-0 (PMC8240300; doi:10.1186/s12931-021-01774-0)

**Methods**

**Patients**

This is a prospective, monocentric, observational study including 30 patients diagnosed with severe asthma based on ERS/ATS guidelines and GINA 2019 criteria, that underwent BT between January 2017 and January 2019 in the University Hospital of Basel. All 30 patients fulfilled the indication for BT as they all had a symptomatic disease with severe, persistent, poorly-controlled symptoms, recurrent exacerbations, emergency department visits and hospitalizations despite maximal medical treatment. Patients were prospectively classified in four groups: 1) asthma patients with high (≥300/μl) blood eosinophils; 2) asthma patients with atopy (IgE≥100 U/ml); 3) asthma patients with allergy (positive prick test to at least one of 16 most common aeroallergens comprising grass, rye, birch, hazelnut tree, alder, ash tree, ribwort, mug wort, ambrosia, *Dermatophagoides pteronyssinus*, *Dermatophagoides farinae,* dog hair, cat hair, *Aspergillus fumigatus*, Cladosporium, Penicillium mix); 4) asthma patients with relevant smoke exposure (≥15 PY).

**Bronchial Thermoplasty (BT)**

All BTs were performed at the bronchoscopy facilities of the University Hospital Basel. Bronchoscopy was performed trans-nasally or trans-orally with the patients in the semi-recumbent position. Nasal anesthesia was achieved by 2% lidocaine gel and the patients received propofol in repeated intravenous boluses or IV perfusion, as previously described (12-14). All patients underwent three sessions of BT separated by at least 1-month intervals (15). During the procedure, EBB specimens were obtained from first- and second- generation bronchi using 2.2 mm wide single use biopsy forceps with Endo-Glide Sheath (Radial Jaw, Boston Scientific). All EBB specimens were washed in PBS, fixed in formalin and transferred to pathology.

**Histological evaluation**

To evade dissimilarities in the histology between different lobes (16), we performed analysis of EBB of individual patients (n=3-5) obtained from the right lower lobe before BT and after each BT. For each specimen, 5 sequential sections were stained with Hematoxylin/ Eosin and Elastica van Gieson and were evaluated blindly by 2 senior pathologists. Only specimens with tangential sections were evaluated, so that the orientation of the sections would not affect the measurements.

Inflammation in the stroma, tissue lymphocyte/ eosinophil/ granulocyte infiltration and thickening of reticular basement membrane (BM), were appraised using a 0-3 scale: 0=absence/normal, 1=mild-moderate, 2-3=severe. The median value of all assessments for these categorical measurements was assigned to each patient as follows: 0-<0.5=(absence/normal), 0.5-<1.50=mild-moderate, 1.5-3=severe.

Airway smooth muscle (ASM) mass was evaluated as the total percentage of the submucosal area occupied by ASMC. The distance between BM and ASM in μm, was measured from the parenchymal site of the BM towards the ASM, without including the thickness of the BM. The mean values of all assessments for these numerical measurements was assigned to each patient.

The scores of both pathologists agreed in more than 95% of the evaluations for each of the 7 parameters that were assessed.

**Immunohistochemistry**

EBB tissue sections obtained before and after BT were enclosed in paraffin blocks. Unstained sections from the blocks were deparaffinised and re-hydrated using a standard protocol. Warm (95°C) citric acid buffer (1M, pH 8.0) was used to unmask the antigens before the slides were blocked with 10% goat serum (Cat. #50062, Thermofisher).

Proliferative epithelial cells and subepithelial mesenchymal cells were assessed using an antibody for Ki67 (Cat. # 550609, BD). The sections were incubated overnight at 4°C with the antibody. Subsequently, biotin-streptavidin-HRP conjugated labelling was performed, followed by DAB-chromogen (brown) staining (Vector Laboratories, USA). Nuclei were counterstained by haematoxylin. Evaluation of cell proliferation was performed by counting cells stained positive for Ki67 in 3 randomly selected areas on each of the slides under the 200X magnification of the microscope (Nikon Eclipse Ti2 inverted microscope system).

Expression of glucocorticoid receptor was assessed using a monoclonal antibody for glucocorticoid receptor (GR) (EPR19621, Abcam, UK) and expression of HSP70 and HSP90 were assessed using specific monoclonal antibodies (for HSP70: cat. #AP-100, for HSP90: cat. # AP-160, Bio-Techne, Zug, Switzerland) following the same procedure as described above.

**Figure legends**

**Additional Figure 1**. Representative microphotographs of endobronchial biopsies obtained from asthma patients before and after thermoplasty, at different magnifications (X100-X400).


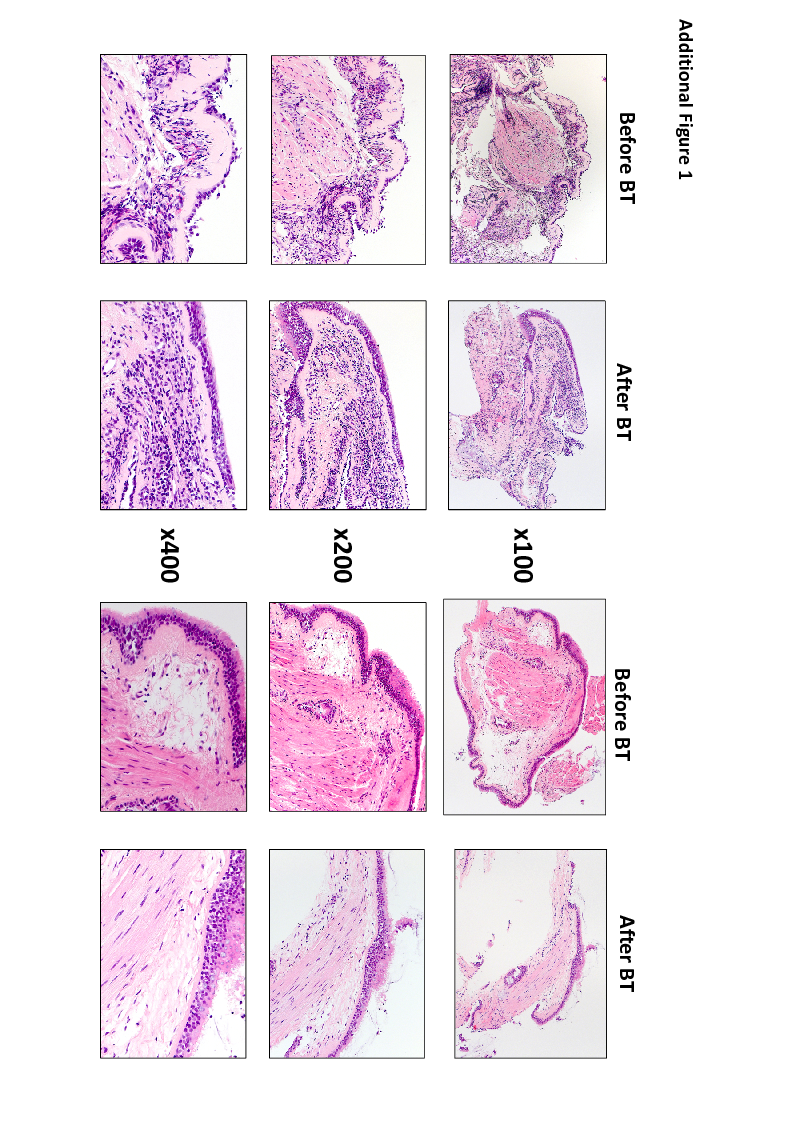

Supplement: Supplementary file 1 — Additional file 1: Figure S1. Representative microphotographs of endobronchial biopsies obtained from asthma patients before and after thermoplasty, at different magnifications (X100-X400). [file 12931_2021_1774_MOESM1_ESM.docx]
