# Supplementary material for: Bronchial thermoplasty in asthma: an exploratory histopathological evaluation in distinct asthma endotypes/phenotypes
Source: Respir Res. 2021 Jun 28;22:186. doi: 10.1186/s12931-021-01774-0 (PMC8240300; doi:10.1186/s12931-021-01774-0)
Supplement: Supplementary file 2 — Additional file 2: Table S1. Lung function parameters in patients with severe asthma after bronchial thermoplasty. [file 12931_2021_1774_MOESM2_ESM.docx]

**Additional Table 1.** Lung function parameters in patients with severe asthma after bronchial thermoplasty

| **Parameter** | **All asthma patients**  **(N=30)** |
| --- | --- |
| **FEV_1_ % of predicted value, mean ± SD** |  |
| Before 1^st^ thermoplasty | 66.94 ± 19.66 |
| After 1^st^ thermoplasty | 68.04 ± 21.41 |
| After 2^nd^ thermoplasty | 69.32 ± 19.37 |
| After 3^rd^ thermoplasty* | 68.43 ± 19.19 |
| **FVC % of predicted value, mean ± SD** |  |
| Before 1^st^ thermoplasty | 94.71 ± 19.11 |
| After 1^st^ thermoplasty | 92.19 ± 21.50 |
| After 2^nd^ thermoplasty | 96.25 ± 16.64 |
| After 3^rd^ thermoplasty* | 95.11 ± 19.06 |
| **FEV_1_/FVC % of predicted value, mean ± SD** |  |
| Before 1^st^ thermoplasty | 73.00 ± 17.69 |
| After 1^st^ thermoplasty | 75.69 ± 16.74 |
| After 2^nd^ thermoplasty | 73.69 ± 15.58 |
| After 3^rd^ thermoplasty* | 74.04 ± 14.80 |
| **RV % of predicted value, mean ± SD** |  |
| Before 1^st^ thermoplasty | 118.49 ± 37.40 |
| After 1^st^ thermoplasty | 115.98 ± 32.33 |
| After 2^nd^ thermoplasty | 118.39 ± 31.84 |
| After 3^rd^ thermoplasty* | 117.75 ± 33.30 |
| **TLC % of predicted value, mean ± SD** |  |
| Before 1^st^ thermoplasty | 104.27 ± 17.07 |
| After 1^st^ thermoplasty | 104.70 ± 15.03 |
| After 2^nd^ thermoplasty | 104.83 ± 14.85 |
| After 3^rd^ thermoplasty* | 104.36 ± 17.23 |
| **RV/TLC % of predicted value, mean ± SD** |  |
| Before 1^st^ thermoplasty | 108.64 ± 22.87 |
| After 1^st^ thermoplasty | 106.82 ± 20.37 |
| After 2^nd^ thermoplasty | 109.21 ± 19.89 |
| After 3^rd^ thermoplasty* | 108.70 ± 22.54 |
| **DLCO % of predicted value, mean ± SD** |  |
| Before 1^st^ thermoplasty | 89.13 ± 20.85 |
| After 1^st^ thermoplasty | 89.07 ± 15.97 |
| After 2^nd^ thermoplasty | 88.62 ± 16.84 |
| After 3^rd^ thermoplasty* | 89.17 ± 18.55 |

*Measurements of lung function parameters were performed after a mean of 49.65 ± 35.25 days after the 3^rd^ thermoplasty. All values shown are post-bronchodilation.

FEV_1_: forced expiratory volume in 1 second; FVC: forced vital capacity; TLC: total lung capacity; RV: residual volume; DLCO: diffusing capacity for carbon monoxide;
