# Supplementary material for: Bronchial thermoplasty in asthma: an exploratory histopathological evaluation in distinct asthma endotypes/phenotypes
Source: Respir Res. 2021 Jun 28;22:186. doi: 10.1186/s12931-021-01774-0 (PMC8240300; doi:10.1186/s12931-021-01774-0)
Supplement: Supplementary file 3 — Additional file 3: Table S2. Association between blood eosinophils and tissue eosinophilic infiltration before BT using generalized linear models. [file 12931_2021_1774_MOESM3_ESM.docx]

**Additional Table 2.** Association between blood eosinophils and tissue eosinophilic infiltration before BT using generalized linear models

| **Time point** | **Factor** | **Value** | **Estimate** | **SD** | **p-value** | **Adjusted Mean** | **95% for the mean** | **p-Value*** | **p-value**** |
| --- | --- | --- | --- | --- | --- | --- | --- | --- | --- |
| Before BT | Intercept | - | 0.219 | 0.053 | 0.0003 |  |  |  |  |
|  | Tissue Eosinophilic Infiltration | No | -0.058 | 0.080 | 0.4745 | 0.1608 | 0.039 - 0.282 | *Ref.* | 0.474 |
|  |  | Yes | 0 |  |  | 0.2186 | 0.109 - 0.328 | 0.4745 |  |
| 1 month after the  1^st^ BT | Intercept | - | 0.211 | 0.071 | 0.0064 |  |  |  |  |
|  | Tissue Eosinophilic Infiltration | No | -0.060 | 0.085 | 0.4871 | 0.1514 | 0.054 - 0.249 | *Ref.* | 0.487 |
|  |  | Yes | 0 |  |  | 0.2114 | 0.065 - 0.357 | 0.4871 |  |
| 1 month after the  2^nd^ BT | Intercept | - | 0.270 | 0.072 | 0.0013 |  |  |  |  |
|  | Tissue Eosinophilic Infiltration | No | -0.1466 | 0.094 | 0.1349 | 0.1242 | -0.001 - 0.249 | *Ref.* | 0.135 |
|  |  | Yes | 0 |  |  | 0.2702 | 0.120 - 0.420 | 0.1349 |  |

*Adjusted for multiple comparisons with the Dunnett-Hsu method.

**for the overall effect of the factor

BT: bronchial thermoplasty; SD: standard deviation
