# Supplementary material for: Bronchial thermoplasty in asthma: an exploratory histopathological evaluation in distinct asthma endotypes/phenotypes
Source: Respir Res. 2021 Jun 28;22:186. doi: 10.1186/s12931-021-01774-0 (PMC8240300; doi:10.1186/s12931-021-01774-0)
Supplement: Supplementary file 4 — Additional file 4: Table S3. Histopathological evaluation of endobronchial biopsies before and after bronchial thermoplasty. [file 12931_2021_1774_MOESM4_ESM.docx]

**Additional Table 3.** Histopathological evaluation of endobronchial biopsies before and after bronchial thermoplasty

|  | **Asthma patients without tissue eosinophilic infiltration before BT (N=14)** | | | **Asthma patients with tissue eosinophilic infiltration before BT (N=16)** | | | **P value**^*^ |
| --- | --- | --- | --- | --- | --- | --- | --- |
|  | **Before**  **1^st^ BT** | **At 2^nd^ BT**  **1 month after**  **1^st^ BT** | **At 3^rd^ BT**  **1 month after**  **2^nd^ BT** | **Before**  **1^st^ BT** | **At 2^nd^ BT**  **1 month after**  **1^st^ BT** | **At 3^rd^ BT**  **1 month after**  **2^nd^ BT** |  |
| **Inflammation in the stroma****  Absence, n (%)  Mild-Moderate, n (%)  High, n (%) | N=14  5 (35.7)  7 (50.0)  2 (14.3) | N=12  4 (33.3)  7 (58.3)  1 (8.3) | N=11  1 (9.1)  8 (72.7)  2 (18.2) | N=16  0  8 (50.0)  8 (50.0) | N=15  1 (6.7)  6 (40.0)  8 (53.3) | N=12  3 (25.0)  4 (33.3)  5 (41.7) | **^0.140^** |
| **Tissue lymphocyte infiltration****  Absence, n (%)  Mild-moderate, n (%)  High, n (%) | N=14  3 (21.4)  8 (57.1)  3 (21.4) | N=12  4 (33.3)  7 (58.3)  1 (8.3) | N=11  1 (9.1)  9 (81.8)  1 (9.1) | N=16  4 (25.0)  4 (25.0)  8 (50.0) | N=15  1 (6.7)  8 (53.3)  6 (40.0) | N=12  3 (25.0)  5 (41.7)  4 (33.3) | **^0.353^** |
| **Granulocytes in the stroma****  Absence, n (%)  Mild-Moderate, n (%)  High, n (%) | N=14  11 (78.6)  3 (21.4)  0 | N=12  10 (83.3)  2 (16.7)  0 | N=11  8 (72.7)  3 (27.3)  0 | N=16  8 (50.0)  8 (50.0)  0 | N=15  11 (73.3)  4 (26.7)  0 | N=12  10 (83.3)  1 (8.3)  1 (8.3) | **^0.620^** |
| **BM thickening ****  Normal, n (%)  Mild-moderate, n (%)  High, n (%) | N=14  1 (7.1)  9 (64.3)  4 (28.6) | N=10  1 (10.0)  8 (80.0)  1 (10.0) | N=11  1 (9.1)  7 (63.6)  3 (27.3) | N=16  0  10 (62.5)  6 (37.5) | N=15  4 (26.7)  7 (46.7)  4 (26.7) | N=12  1 (8.3  7 (58.3)  4 (33.3) | **^0.927^** |
| **Average ASM mass (%)**  Median [IQR]  Mean (SD) | N=14  6.9 [0.8-23.9]  14.3 (18.1) | N=11  2.5 [0.0-12.5]  8.7 (11.5) | N=11  1.0 [0.0-2.5]  5.5 (10.8) | N=16  19.2 [11.0-33.7]  23.1 (16.4) | N=15  5.0 [0.0-15.0]  12.2 (17.0) | N=12  4.2 [0.0-20.0]  13.4 (20.0) | **^0.833^** |
| **Distance BM-ASM** μm  Median [IQR]  Mean (SD) | N=9  71.5 [39.2-127.7]  95.0 (72.6) | N=2  82.9 [54.5-]  82.9 (40.2) | N=3  87.4 [32.6-]  127.0 (119.2) | N=15  73.5 [33.9-96.0]  77.2 (45.1) | N=9  56.5 [22.4-165.4]  97.7 (94.8) | N=5  75.8 [30.7-122.3]  76.4 (47.1) | **^0.457^** |

* Comparisons were made for the changes in various parameters between asthma patients with tissue eosinophilic infiltration and patients without tissue eosinophilic infiltration. The p-value was calculated by introducing the interaction between time of BT and the classification factor in multinomial logistic regression model for categorical variables and a mixed linear regression for continuous variables.

** qualitative evaluation, 0 to 3 scale: 0 to <0.5 = absence / normal, 0.5 to <1.50 = mild-moderate, 1.5 to 3= severe

BT: bronchial thermoplasty; BM: basement membrane; ASM: airway smooth muscle; IQR: inter quartile range; SD: standard deviation
